# Changes in Alcohol Consumption and Risk of Dementia in a Nationwide Cohort in South Korea

**DOI:** 10.1001/jamanetworkopen.2022.54771

**Published:** 2023-02-06

**Authors:** Keun Hye Jeon, Kyungdo Han, Su-Min Jeong, Junhee Park, Jung Eun Yoo, Juhwan Yoo, Jinkook Lee, SangYun Kim, Dong Wook Shin

**Affiliations:** 1 Department of Family Medicine, Cha Gumi Medical Center, Cha University, Gumi, Republic of Korea; 2 Department of Statistics and Actuarial Science, Soongsil University, Seoul, Republic of Korea; 3 Department of Medicine, Seoul National University College of Medicine, Seoul, Republic of Korea; 4 Department of Family Medicine, Seoul National University Health Service Center, Seoul, Republic of Korea; 5 Department of Family Medicine, Seoul National University Hospital, Seoul, Republic of Korea; 6 Department of Family Medicine/Supportive Care Center, Samsung Medical Center, Sungkyunkwan University School of Medicine, Seoul, Republic of Korea; 7 Department of Family Medicine, Healthcare System Gangnam Center Seoul National University Hospital, Seoul, Republic of Korea; 8 Department of Medical Statistics, The Catholic University of Korea, Seoul, Republic of Korea; 9 Department of Economics, Center for Economic & Social Research, University of Southern California, Los Angeles; 10 RAND Corporation, Santa Monica, California; 11 Department of Neurology, Seoul National University Bundang Hospital & Seoul National University College of Medicine, Seongnam, Republic of Korea; 12 Department of Clinical Research Design and Evaluation, Samsung Advanced Institute for Health Science and Technology, Sungkyunkwan University, Seoul, Republic of Korea

## Abstract

**Importance:**

The impact of serial changes in alcohol consumption on dementia risk has rarely been investigated to date.

**Objective:**

To investigate the association of comprehensive patterns of changes in alcohol consumption with the incidence of all-cause dementia, Alzheimer disease (AD), and vascular dementia (VaD).

**Design, Setting, and Participants:**

This is a retrospective cohort study. Data were obtained from the Korean National Health Insurance Service database. Adults aged 40 years and older underwent 2 health examinations in 2009 and 2011. The cohort was assessed until December 31, 2018, and statistical analysis was performed in December 2021.

**Exposures:**

Alcohol consumption level was categorized into none (0 g per day), mild (<15 g per day), moderate (15-29.9 g per day), and heavy (≥30 g per day) drinking. On the basis of changes in alcohol consumption level from 2009 to 2011, participants were categorized into the following groups: nondrinker, quitter, reducer, sustainer, and increaser.

**Main Outcomes and Measures:**

The primary outcome was newly diagnosed AD, VaD, or other dementia.

**Results:**

Among 3 933 382 participants (mean [SD] age, 55.0 [9.6] years; 2 037 948 men [51.8%]), during a mean (SD) follow-up of 6.3 (0.7) years, there were 100 282 cases of all-cause dementia, 79 982 cases of AD, and 11 085 cases of VaD. Compared with sustained nondrinking, sustained mild (adjusted hazard ratio [aHR], 0.79; 95% CI, 0.77-0.81) and moderate (aHR, 0.83; 95% CI, 0.79-0.88) drinking were associated with a decreased risk of all-cause dementia, whereas sustained heavy drinking was associated with an increased risk of all-cause dementia (aHR, 1.08; 95% CI, 1.03-1.12). Compared with sustained levels of drinking, reducing alcohol consumption from a heavy to a moderate level (aHR, 0.92; 95% CI, 0.86-0.99) and the initiation of mild alcohol consumption (aHR, 0.93; 95% CI, 0.90-0.96) were associated with a decreased risk of all-cause dementia. Increasers and quitters exhibited an increased risk of all-cause dementia compared with sustainers. The trends in AD and VaD remained consistent.

**Conclusions and Relevance:**

In this cohort study of a Korean population, decreased risk of dementia was associated with maintaining mild to moderate alcohol consumption, reducing alcohol consumption from a heavy to a moderate level, and the initiation of mild alcohol consumption, suggesting that the threshold of alcohol consumption for dementia risk reduction is low.

## Key Points

**Question:**

Is a change in alcohol consumption associated with the incidence of dementia?

**Findings:**

In this cohort study of 3 933 382 individuals in Korea, maintaining mild to moderate alcohol consumption was associated with a decreased risk of dementia compared with sustained nondrinking, whereas sustained heavy drinking of alcohol was associated with an increased risk of dementia. Reduction of drinking from a heavy to a moderate level and initiation of mild drinking were associated with a decreased risk of dementia compared with a sustained level of drinking.

**Meaning:**

These findings suggest that the threshold of alcohol consumption for dementia risk reduction is low.

## Teaser

This cohort study investigates the association of comprehensive patterns of changes in alcohol consumption with the incidence of all-cause dementia, Alzheimer disease, and vascular dementia among individuals in Korea.

## Introduction

Currently, more than 57 million people live with dementia worldwide, and this number is expected to increase to more than 152 million by 2050.^1^ Alcohol consumption is generally considered as a potential modifiable risk factor for dementia, but the results in the literature are not completely consistent.^2^ Several longitudinal studies^3,4,5,6^ reported an association of mild to moderate alcohol consumption with a reduced risk of dementia, whereas others showed no association.^7,8,9,10^ Notably, most studies assessed alcohol consumption only once at baseline. Only a few studies considered a change of alcohol consumption during the study period and its association with the incidence of dementia (eTable 1 in Supplement 1). Sabia et al^4^ examined the link between 17-year trajectories of alcohol consumption and the risk of dementia in the UK. The authors concluded that the risk of dementia was increased in those with long-term abstinence, decreased alcohol consumption, and long-term consumption greater than 14 units per week compared with participants with long-term consumption of 1 to 14 units per week.^4^ However, that study did not conduct stratified analyses considering the initial amount of alcohol consumption level (eg, mild, moderate, or heavy). Mukamal et al^5^ examined alcohol consumption in 2 separate assessments. In that study, however, the participants were simply categorized according to the average level of alcohol consumption between 2 measurements (eg, <1 drinks per week) without reflecting changes in the pattern (eg, decreased or increased) of alcohol consumption between the measurements.^5^ In addition, that study used only the abstainers, but not the sustainers, as a reference, making it difficult to accurately reflect the effect of changes in the pattern of alcohol consumption on the risk of dementia.

In the present study, we evaluated the association between comprehensive patterns of changes in alcohol consumption and the risk of dementia stratified by the initial amount of alcohol consumption using a large sample of a representative Korean population. Notably, to our knowledge, our study is the first to use the sustainers at the same level of alcohol consumption, in addition to the abstainers, as a reference group within each baseline alcohol consumption level, which enables more comprehensive understanding on the association between changes in the pattern of alcohol consumption and risk of dementia.

## Methods

### Data Source and Study Setting

The Korean National Health Insurance Service (NHIS) is a single insurer administered by the Korean government. The NHIS provides a free biennial cardiovascular health examination to all insured individuals aged 40 years and older. The health examination consists of a standard questionnaire regarding medical history and lifestyle habits (eg, drinking, smoking, and exercise), anthropometric measurements, and laboratory tests.^11^ The NHIS also retains qualification data regarding demographics and diagnosis codes for diagnoses and utilization of inpatient and outpatient medical services for insurance claims. This study was approved by the institutional review board of the Samsung Medical Center, which waived the need for informed consent because the data were publicly available and anonymized under confidentiality guidelines. This study was designed and conducted according to the Strengthening the Reporting of Observational Studies in Epidemiology (STROBE) reporting guideline.

### Study Population

From the NHIS database of the entire Korean population, we collected data of participants aged 40 years and older who had undergone 2 national health examinations in 2009 (first examination) and 2011 (second examination). Among 4 961 817 participants, those who had a previous diagnosis of dementia (11 337 participants), cancer (142 259 participants), or cardiovascular disease (402 191 participants) before their second examination were excluded. In addition, participants who received a diagnosis of dementia (6378 participants), cancer (45 372 participants), or cardiovascular disease (47 187 participants) and those who died (5373 participants) within 1 year after their second examination (called the 1-year lag period) were excluded to minimize possible reverse causality. Finally, those whose records were missing any information on alcohol consumption or other key variables (368 338 participants) were excluded. We also established a subgroup of people who participated in 3 consecutive health examinations to assess the impact of further changes in drinking level at a third examination in 2013. A total of 2 977 137 individuals were included in this subgroup analysis (eFigure 1 in Supplement 1).

### Alcohol Intake

Information on alcohol consumption was obtained from self-reported questionnaires regarding the frequency (the number of days per week) and quantity (the number of standard drinks on each occasion) of alcohol consumption in the past 12 months. The number of standard drinks was converted to measurements of pure alcohol in grams, which is approximately 8 g for a typical volume of beer, wine, soju (Korean traditional alcohol), or whisky.^12^ The weekly frequency and pure alcohol amount per occasion were multiplied to calculate the total amount of alcohol consumption per week, which was then converted to the daily amount of alcohol intake. The participants were classified into 4 groups: none (0 g per day), mild (<15 g per day), moderate (15-29.9 g per day), or heavy (≥30 g per day), according to the Dietary Guidelines for Americans.^13^ The participants were then assigned to 1 of 5 groups according to change in alcohol consumption from 2009 to 2011: (1) sustained nondrinkers, (2) quitters (those who stopped drinking), (3) reducers (those who reduced their level of consumption but did not stop drinking), (4) sustainers (those who maintained the same level of consumption), and (5) increasers (those who increased their level of consumption). Information on covariates is described in the eMethods in Supplement 1.

### Study Outcomes and Follow-up

The end points of the study were newly diagnosed dementia, which was identified by new claims with the *International Statistical Classification of Diseases and Related Health Problems, Tenth Revision (ICD-10)* codes for AD (*ICD-10* codes F00 or G30), VaD (*ICD-10* code F01), or other dementia (*ICD-10* codes F02, F03, G23.1, or G31) combined with the prescription of antidementia drugs at least twice. To file expense claims for the prescription of acetylcholinesterase inhibitors (donepezil hydrochloride, rivastigmine, and galantamine) or the N-methyl-D-aspartate receptor antagonist (memantine) for dementia treatment, physicians need to document evidence of cognitive dysfunction according to National Health Insurance Reimbursement criteria: a Mini-Mental State Examination score of 26 or less and either a Clinical Dementia Rating of 1 or higher or a Global Deterioration Scale score of 3 or higher.^14,15,16^ The cohort was assessed from 1 year after the second health examination to the date of incident dementia or death, or until the end of the study period (December 31, 2018), whichever came first.

### Statistical Analysis

Continuous variables were presented as mean (SD), and categorical variables were presented as number and percentage. Cox proportional hazard regression analyses were conducted to estimate hazard ratios (HRs) and 95% CIs for the association of change in alcohol consumption with incidence of dementia. To determine which covariates to include in multivariable-adjusted proportional hazards models, we used a directed acyclic diagram^17^ (eFigure 2 in Supplement 1). Model 1 was adjusted for age, sex, smoking status, regular exercise, area of residence, and income. Model 2 was additionally adjusted for comorbidities (hypertension, diabetes, and dyslipidemia), body mass index (calculated as weight in kilograms divided by height in meters squared), systolic blood pressure, and laboratory results (fasting glucose levels, total cholesterol, and serum creatinine). Comparisons were done using 2 different reference groups: (1) sustained nondrinkers as a reference group to compare the association across all categories and (2) sustainers as a reference group within each baseline category.

Sensitivity analysis with competing risk analysis was performed with the Fine and Gray method to assess the subdistribution HR for dementia incidence considering death from any cause as a competing event. Stratified analyses were performed by age (65 years old), sex, and smoking status. Statistical analyses were performed using SAS statistical software version 9.4 (SAS Institute), and 2-sided *P* < .05 was considered statistically significant. Statistical analysis was performed in December 2021.

## Results

### Baseline Characteristics

After exclusions, a total of 3 933 382 participants (mean [SD] age, 55.0 [9.6] years; 2 037 948 men [51.8%]) were included in our analyses. At the first examination, 2 157 126 participants (54.8%) were nondrinkers, 1 048 578 (26.7%) were mild drinkers, 431 203 (11.0%) were moderate drinkers, and 296 475 (7.5%) were heavy drinkers (eTable 2 in Supplement 1). From 2009 to 2011, 253 643 mild drinkers (24.2%), 36 329 moderate drinkers (8.4%), and 22 604 heavy drinkers (7.6%) became quitters, whereas 299 206 nondrinkers (13.9%), 169 212 mild drinkers (16.1%), and 75 124 moderate drinkers (17.4%) increased their drinking level (eTable 3 in Supplement 1). Table 1 (total study population) and eTable 4 in Supplement 1 (divided by sex) show the baseline characteristics according to changes in alcohol consumption. Sustained nondrinkers had the oldest mean age and the highest proportion of female participants (1 380 847 participants [74.3%]) among the 5 categories and were mostly nonsmokers (1 572 583 participants [84.6%]). Compared with sustainers, quitters tended to be older, female, nonsmokers, be more engaged in regular exercise, and have lower incomes.

**Table 1.** Baseline 2011 Characteristics of the Study Population by Change in Daily Amount of Alcohol Consumption Between 2009 and 2011

Variable	Participants, No. (%)				
	Nondrinkers (n = 1 857 920)	Quitters (n = 312 576)	Reducers (n = 284 205)	Sustainers (n = 935 139)	Increasers (n = 543 542)
Age, mean (SD), y	57.3 (10.0)	54.8 (9.6)	52.8 (8.6)	52.2 (8.4)	53.1 (8.9)
Sex					
Male	477 073 (25.7)	172 112 (55.1)	258 738 (91.0)	741 691 (79.3)	388 334 (71.5)
Female	1 380 847 (74.3)	140 464 (44.9)	25 467 (9.0)	193 448 (20.7)	155 208 (28.5)
Alcohol consumption status in 2009					
Nondrinker	1 857 920 (100)	NA	NA	NA	299 206 (55.1)
Mild (<15 g/d)	NA	253 643 (81.1)	NA	625 723 (66.9)	169 212 (31.1)
Moderate (15-29 g/d)	NA	36 329 (11.6)	151 249 (53.2)	168 501 (18.0)	75 124 (13.8)
Heavy (≥30 g/d)	NA	22 604 (7.2)	132 956 (46.8)	140 915 (15.1)	NA
Smoking status					
None	1 572 583 (84.6)	219 395 (70.2)	74 338 (26.2)	350 751 (37.5)	238 293 (43.8)
Former	148 211 (8.0)	47 328 (15.1)	89 799 (31.6)	276 834 (29.6)	139 242 (25.6)
Current smoker, cigarettes/d					
<10	17 405 (0.9)	6504 (2.1)	12 629 (4.4)	32 993 (3.5)	17 325 (3.2)
10-19	50 850 (2.7)	17 685 (5.7)	48 041 (16.9)	123 503 (13.2)	60 875 (11.2)
≥20	68 871 (3.7)	21 664 (6.9)	59 398 (20.9)	151 058 (16.2)	87 807 (16.2)
Regular physical activity					
No	1 481 916 (79.8)	248 680 (79.6)	213 679 (75.2)	705 441 (75.4)	412 993 (76.0)
Yes	376 004 (20.2)	63 896 (20.4)	70 526 (24.8)	229 698 (24.6)	130 549 (24.0)
Anthropometric measures, mean (SD)					
Body mass index^a^	23.8 (3.1)	24.0 (3.0)	24.3 (2.9)	24.0 (2.8)	24.1 (2.9)
Waist circumference, cm	79.6 (8.5)	81.1 (8.5)	84.1 (7.6)	82.5 (8.0)	82.4 (8.3)
Systolic blood pressure, mm Hg	122.9 (15.2)	123.0 (14.8)	126.4 (14.3)	124.6 (14.3)	124.7 (14.7)
Diastolic blood pressure, mm Hg	75.8 (9.8)	76.6 (9.8)	79.4 (9.8)	78.2 (9.8)	78.1 (10.0)
Comorbidities					
Hypertension	637 346 (34.3)	102 292 (32.7)	105 828 (37.2)	301 064 (32.2)	181 570 (33.4)
Diabetes	204 017 (11.0)	35 245 (11.3)	37 329 (13.1)	98 467 (10.5)	62 038 (11.4)
Dyslipidemia	485 111(26.1)	70 406 (22.5)	60 048 (21.1)	185 446 (19.8)	111 455 (20.5)
Chronic kidney disease	128 245 (6.9)	16 254 (5.2)	11 149 (3.9)	37 839 (4.1)	22 970 (4.2)
Laboratory findings, mean (SD)					
Glucose, mg/dL	98.0 (22.3)	99.4 (23.8)	103.0 (25.9)	100.7 (23.7)	101.0 (24.9)
Total cholesterol, mg/dL	199.7 (37.0)	197.7 (36.6)	197.7 (35.7)	197.9 (35.1)	198.1 (35.7)
High-density lipoprotein cholesterol, mg/dL	54.7 (16.6)	54.5 (17.3)	54.8 (17.4)	55.3 (17.4)	55.9 (18.3)
Low-density lipoprotein cholesterol, mg/dL	120.5 (33.9)	117.7 (33.9)	112.3 (34.7)	114.6 (33.8)	114.4 (34.1)
Glomerular filtration rate, mL/min/1.73 m^2^	86.8 (30.9)	87.8 (32.5)	88.9 (38.0)	88.3 (37.5)	88.6 (35.1)
Urban residency	818 363 (44.1)	140 824 (45.1)	129 769 (45.7)	444 138 (47.5)	245 770 (45.2)
Income level by quartile					
1 (Lowest)	486 229 (26.2)	75 740 (24.2)	51 836 (18.2)	175 305 (18.8)	116 334 (21.4)
2	351 457 (18.9)	60 273 (19.3)	47 283 (16.6)	153 620 (16.4)	96 307 (17.7)
3	424 495 (22.9)	73 060 (23.4)	71 949 (25.3)	220 614 (23.6)	129 002 (23.7)
4 (Highest)	595 739 (32.1)	103 503 (33.1)	113 137 (39.8)	385 600 (41.2)	201 899 (37.2)

Abbreviation: NA, not applicable.

SI conversion factors: To convert glucose to millimoles per liter, multiply by 0.0555; high-density lipoprotein cholesterol to millimoles per liter, multiply by 0.0259; low-density lipoprotein cholesterol to millimoles per liter, multiply by 0.0259; total cholesterol to millimoles per liter, multiply by 0.0259.

^a^ Body mass index is calculated as weight in kilograms divided by height in meters squared.

### Alcohol Consumption and Dementia

During a mean (SD) follow-up of 6.3 (0.7) years after a 1-year lag period, there were 100 282 cases (2.5%) of all-cause dementia, 79 982 cases (2.0%) of AD, and 11 085 cases (0.3%) of VaD. Compared with sustained nondrinkers, those who sustained mild or moderate alcohol consumption had a significantly lower risk of all-cause dementia (21% lower for mild to mild, adjusted HR [aHR], 0.79; 95% CI, 0.77-0.81; 17% lower for moderate to moderate, aHR, 0.83; 95% CI, 0.79-0.88), whereas sustained heavy drinkers had an 8% higher risk of all-cause dementia (aHR, 1.08; 95% CI, 1.03-1.12) (Table 2). Similar patterns were also observed in both AD and VaD. The association was smaller for VaD than AD possibly because the smaller number of cases of VaD (11 085 participants) compared with cases of AD (79 982 participants).

**Table 2.** HRs and 95% CIs for the Association of Change in Alcohol Consumption Amount With Risk of Dementia, With Nondrinkers as the Reference

Type of dementia and alcohol consumption status in 2009 and 2011	Participants, No.	Events, No.	IR/1000 PYs	Crude model		Model 1^a^		Model 2^b^	
				HR (95% CI)	*P* value	HR (95% CI)	*P* value	HR (95% CI)	*P* value
All-cause dementia									
Nondrinker in 2009					<.001		<.001		<.001
Nondrinker in 2011	1 857 920	68 679	5.9	1 [Reference]		1 [Reference]		1 [Reference]	
Mild in 2011	243 958	4338	2.8	0.48 (0.47-0.50)		0.93 (0.90-0.95)		0.93 (0.90-0.96)	
Moderate in 2011	34 821	718	3.3	0.57 (0.53-0.61)		1.13 (1.05-1.22)		1.11 (1.03-1.20)	
Heavy in 2011	20 427	556	4.4	0.75 (0.69-0.81)		1.33 (1.22-1.44)		1.30 (1.19-1.41)	
Mild in 2009									
Nondrinker in 2011	253 643	6153	3.9	0.66 (0.64-0.68)		1.02 (0.99-1.05)		1.02 (0.99-1.05)	
Mild in 2011	625 723	6690	1.7	0.29 (0.28-0.30)		0.78 (0.76-0.81)		0.79 (0.77-0.81)	
Moderate in 2011	130 116	1471	1.8	0.31 (0.29-0.33)		0.86 (0.81-0.91)		0.86 (0.82-0.91)	
Heavy in 2011	39 096	767	3.1	0.54 (0.50-0.58)		1.11 (1.04-1.20)		1.10 (1.02-1.18)	
Moderate in 2009									
Nondrinker in 2011	36 329	1060	4.7	0.79 (0.75-0.84)		1.23 (1.16-1.31)		1.23 (1.16-1.31)	
Mild in 2011	151 249	1929	2.0	0.35 (0.34-0.37)		0.92 (0.88-0.97)		0.92 (0.88-0.96)	
Moderate in 2011	168 501	1521	1.4	0.25 (0.24-0.26)		0.84 (0.79-0.88)		0.83 (0.79-0.88)	
Heavy in 2011	75 124	986	2.1	0.36 (0.34-0.38)		0.99 (0.93-1.05)		0.98 (0.92-1.04)	
Heavy in 2009									
Nondrinker in 2011	22 604	883	6.3	1.09 (1.02-1.16)		1.45 (1.35-1.55)		1.42 (1.33-1.52)	
Mild in 2011	49 657	1005	3.2	0.56 (0.52-0.59)		1.13 (1.06-1.20)		1.12 (1.06-1.20)	
Moderate in 2011	83 299	1153	2.2	0.38 (0.36-0.41)		0.99 (0.94-1.06)		0.98 (0.93-1.05)	
Heavy in 2011	140 915	2373	2.7	0.46 (0.44-0.48)		1.09 (1.04-1.14)		1.08 (1.03-1.12)	
Alzheimer disease									
Nondrinker in 2009					<.001		<.001		<.001
Nondrinker in 2011	1 857 920	55 823	4.8	1 [Reference]		1 [Reference]		1 [Reference]	
Mild in 2011	243 958	3416	2.2	0.47 (0.46-0.49)		0.93 (0.89-0.96)		0.93 (0.90-0.96)	
Moderate in 2011	34 821	559	2.6	0.55 (0.50-0.59)		1.14 (1.05-1.24)		1.13 (1.04-1.23)	
Heavy in 2011	20 427	427	3.4	0.70 (0.63-0.77)		1.31 (1.18-1.44)		1.30 (1.18-1.43)	
Mild in 2009									
Nondrinker in 2011	253 643	4817	3.0	0.63 (0.62-0.65)		1.00 (0.97-1.04)		1.01 (0.98-1.04)	
Mild in 2011	625 723	5066	1.3	0.27 (0.26-0.28)		0.77 (0.75-0.80)		0.78 (0.75-0.80)	
Moderate in 2011	130 116	1104	1.3	0.29 (0.27-0.31)		0.85 (0.80-0.91)		0.85 (0.80-0.91)	
Heavy in 2011	39 096	574	2.3	0.50 (0.46-0.55)		1.09 (1.01-1.19)		1.08 (0.99-1.17)	
Moderate in 2009									
Nondrinker in 2011	36 329	832	3.7	0.76 (0.71-0.82)		1.23 (1.15-1.32)		1.24 (1.15-1.33)	
Mild in 2011	151 249	1474	1.5	0.33 (0.31-0.35)		0.93 (0.88-0.98)		0.92 (0.88-0.98)	
Moderate in 2011	168 501	1072	1.0	0.21 (0.20-0.23)		0.78 (0.73-0.83)		0.79 (0.74-0.84)	
Heavy in 2011	75 124	724	1.5	0.32 (0.30-0.35)		0.95 (0.88-1.03)		0.95 (0.88-1.03)	
Heavy in 2009									
Nondrinker in 2011	22 604	683	4.9	1.05 (0.97-1.13)		1.45 (1.34-1.56)		1.41 (1.31-1.52)	
Mild in 2011	49 657	783	2.5	0.53 (0.49-0.57)		1.14 (1.06-1.23)		1.15 (1.07-1.23)	
Moderate in 2011	83 299	831	1.6	0.34 (0.32-0.36)		0.94 (0.88-1.01)		0.94 (0.88-1.01)	
Heavy in 2011	140 915	1797	2.0	0.43 (0.41-0.45)		1.09 (1.03-1.14)		1.08 (1.03-1.13)	
Vascular dementia									
Nondrinker in 2009					<.001		<.001		<.001
Nondrinker in 2011	1 857 920	6791	0.6	1 [Reference]		1 [Reference]		1 [Reference]	
Mild in 2011	243 958	507	0.3	0.56 (0.51-0.62)		0.93 (0.85-1.03)		0.95 (0.86-1.04)	
Moderate in 2011	34 821	90	0.4	0.72 (0.59-0.89)		1.14 (0.92-1.40)		1.07 (0.87-1.32)	
Heavy in 2011	20 427	69	0.5	0.97 (0.77-1.23)		1.35 (1.06-1.72)		1.21 (0.96-1.54)	
Mild in 2009									
Nondrinker in 2011	253 643	723	0.5	0.79 (0.73-0.85)		1.10 (1.01-1.19)		1.09 (1.01-1.18)	
Mild in 2011	625 723	922	0.2	0.41 (0.38-0.44)		0.84 (0.78-0.91)		0.84 (0.78-0.90)	
Moderate in 2011	130 116	219	0.3	0.46 (0.40-0.53)		0.92 (0.80-1.07)		0.90 (0.79-1.04)	
Heavy in 2011	39 096	120	0.5	0.84 (0.70-1.01)		1.32 (1.09-1.59)		1.27 (1.05-1.52)	
Moderate in 2009									
Nondrinker in 2011	36 329	140	0.6	1.07 (0.91-1.27)		1.40 (1.18-1.66)		1.35 (1.14-1.60)	
Mild in 2011	151 249	260	0.3	0.49 (0.43-0.55)		0.94 (0.83-1.07)		0.89 (0.78-1.01)	
Moderate in 2011	168 501	295	0.3	0.49 (0.44-0.55)		1.14 (1.01-1.29)		1.06 (0.94-1.20)	
Heavy in 2011	75 124	159	0.3	0.58 (0.50-0.69)		1.15 (0.98-1.36)		1.08 (0.92-1.27)	
Heavy in 2009									
Nondrinker in 2011	22 604	109	0.8	1.34 (1.10-1.62)		1.50 (1.23-1.83)		1.46 (1.21-1.77)	
Mild in 2011	49 657	135	0.4	0.79 (0.66-0.93)		1.22 (1.03-1.46)		1.12 (0.94-1.33)	
Moderate in 2011	83 299	202	0.4	0.68 (0.59-0.78)		1.28 (1.11-1.48)		1.19 (1.03-1.38)	
Heavy in 2011	140 915	344	0.4	0.67 (0.60-0.75)		1.16 (1.03-1.30)		1.09 (0.97-1.22)	

Abbreviations: HR, hazard ratio; IR, incidence rate; PY, person-year.

^a^ Model 1 was adjusted for age, sex, smoking status, physical activity, area of residence, and income.

^b^ Model 2 included model 1 plus body mass index, hypertension, diabetes, dyslipidemia, systolic blood pressure, fasting glucose level, total cholesterol level, and serum creatinine.

### Alcohol Consumption Change and Dementia

When sustainers at the same level of alcohol consumption were used as the reference group, nondrinkers who initiated drinking to a mild level were at lower risk for all-cause dementia (aHR, 0.93; 95% CI, 0.90-0.96) and AD (aHR, 0.92; 95% CI, 0.89-0.95) (Table 3 and Figure). We also found that those who reduced their drinking from heavy to moderate levels had a lower risk of all-cause dementia (8% decreased risk; aHR, 0.92; 95% CI, 0.86-0.99) and AD (12% decreased risk; aHR, 0.88; 95% CI, 0.81-0.95). In contrast, increasers exhibited increased risk of all-cause dementia (mild to moderate, aHR, 1.09; 95% CI, 1.03-1.15; mild to heavy, aHR, 1.37; 95% CI, 1.27-1.47; and moderate to heavy, aHR, 1.16; 95% CI 1.07-1.25). Quitters from any level of alcohol consumption showed higher risk of all-cause dementia compared with those who sustained the same level of drinking. The results of the competing analysis (eTable 5 in Supplement 1) and analyses stratified by age, sex, and smoking status (eTables 6, 7, 8, and 9 in Supplement 1) were consistent with those of the main analyses.

**Table 3.** HR and 95% CIs for the Association of Change in Alcohol Consumption Amount With Risk of Dementia, With Sustained Drinking at the Same Level as a Reference

Type of dementia and alcohol consumption status in 2009 and 2011	Participants, No.	Events, No.	IR/1000 PYs	Crude Model		Model 1^a^		Model 2^b^	
				HR (95% CI)	*P* value	HR (95% CI)	*P* value	HR (95% CI)	*P* value
All-cause dementia									
Nondrinker in 2009									
Nondrinker in 2011	1 857 920	68 679	5.9	1 [Reference]	<.001	1 [Reference]	<.001	1 [Reference]	<.001
Mild in 2011	243 958	4338	2.8	0.48 (0.47-0.50)		0.92 (0.89-0.95)		0.93 (0.90-0.96)	
Moderate in 2011	34 821	718	3.3	0.57 (0.53-0.61)		1.13 (1.05-1.22)		1.11 (1.03-1.20)	
Heavy in 2011	20 427	556	4.4	0.75 (0.68-0.81)		1.32 (1.22-1.44)		1.30 (1.19-1.41)	
Mild in 2009									
Nondrinker in 2011	253 643	6153	3.9	2.26 (2.18-2.34)	<.001	1.29 (1.24-1.34)	<.001	1.27 (1.23-1.32)	<.001
Mild in 2011	625 723	6690	1.7	1 [Reference]		1 [Reference]		1 [Reference]	
Moderate in 2011	130 116	1471	1.8	1.06 (1.00-1.12)		1.10 (1.04-1.16)		1.09 (1.03-1.15)	
Heavy in 2011	39 096	767	3.1	1.86 (1.72-2.01)		1.41 (1.31-1.52)		1.37 (1.27-1.47)	
Moderate in 2009									
Nondrinker in 2011	36 329	1060	4.7	3.20 (2.95-3.47)	<.001	1.44 (1.32-1.57)	<.001	1.44 (1.33-1.57)	<.001
Mild in 2011	151 249	1929	2.0	1.42 (1.33-1.52)		1.09 (1.02-1.17)		1.09 (1.02-1.17)	
Moderate in 2011	168 501	1521	1.4	1 [Reference]		1 [Reference]		1 [Reference]	
Heavy in 2011	75 124	986	2.1	1.45 (1.34-1.57)		1.17 (1.08-1.27)		1.16 (1.07-1.25)	
Heavy in 2009									
Nondrinker in 2011	22 604	883	6.3	2.35 (2.17-2.54)	<.001	1.34 (1.23-1.45)	<.001	1.32 (1.22-1.43)	<.001
Mild in 2011	49 657	1005	3.2	1.20 (1.12-1.30)		1.04 (0.96-1.12)		1.05 (0.97-1.13)	
Moderate in 2011	83 299	1153	2.2	0.83 (0.77-0.89)		0.91 (0.85-0.98)		0.92 (0.86-0.99)	
Heavy in 2011	140 915	2373	2.7	1 [Reference]		1 [Reference]		1 [Reference]	
Alzheimer disease									
Nondrinker in 2009									
Nondrinker in 2011	1 857 920	55 823	4.8	1 [Reference]	<.001	1 [Reference]	<.001	1 [Reference]	<.001
Mild in 2011	243 958	3416	2.2	0.47 (0.46-0.49)		0.92 (0.89-0.95)		0.92 (0.89-0.95)	
Moderate in 2011	34 821	559	2.6	0.55 (0.50-0.59)		1.13 (1.04-1.23)		1.12 (1.03-1.21)	
Heavy in 2011	20 427	427	3.4	0.70 (0.63-0.77)		1.30 (1.18-1.43)		1.29 (1.18-1.43)	
Mild in 2009									
Nondrinker in 2011	253 643	4817	3.0	2.33 (2.24-2.43)	<.001	1.27 (1.22-1.32)	<.001	1.26 (1.20-1.31)	<.001
Mild in 2011	625 723	5066	1.3	1 [Reference]		1 [Reference]		1 [Reference]	
Moderate in 2011	130 116	1104	1.3	1.06 (0.99-1.13)		1.12 (1.04-1.19)		1.10 (1.03-1.18)	
Heavy in 2011	39 096	574	2.3	1.84 (1.69-2.01)		1.40 (1.29-1.53)		1.37 (1.25-1.49)	
Moderate in 2009									
Nondrinker in 2011	36 329	832	3.7	3.57 (3.25-3.91)	<.001	1.51 (1.37-1.66)	<.001	1.50 (1.37-1.65)	<.001
Mild in 2011	151 249	1474	1.5	1.55 (1.43-1.67)		1.16 (1.07-1.26)		1.15 (1.06-1.25)	
Moderate in 2011	168 501	1072	1.0	1 [Reference]		1 [Reference]		1 [Reference]	
Heavy in 2011	75 124	724	1.5	1.51 (1.37-1.67)		1.20 (1.09-1.32)		1.18 (1.07-1.30)	
Heavy in 2009									
Nondrinker in 2011	22 604	683	4.9	2.41 (2.20-2.64)	<.001	1.33 (1.21-1.46)	<.001	1.30 (1.18-1.42)	<.001
Mild in 2011	49 657	783	2.5	1.23 (1.13-1.34)		1.05 (0.96-1.14)		1.06 (0.98-1.16)	
Moderate in 2011	83 299	831	1.6	0.78 (0.72-0.85)		0.87 (0.80-0.94)		0.88 (0.81-0.95)	
Heavy in 2011	140 915	1797	2.0	1 [Reference]		1 [Reference]		1 [Reference]	
Vascular dementia									
Nondrinker in 2009									
Nondrinker in 2011	1 857 920	6791	0.6	1 [Reference]	<.001	1 [Reference]	.02	1 [Reference]	.08
Mild in 2011	243 958	507	0.3	0.56 (0.51-0.62)		0.95 (0.86-1.04)		0.95 (0.87-1.05)	
Moderate in 2011	34 821	90	0.4	0.72 (0.59-0.89)		1.16 (0.94-1.43)		1.09 (0.88-1.34)	
Heavy in 2011	20 427	69	0.5	0.97 (0.77-1.23)		1.38 (1.08-1.75)		1.23 (0.97-1.57)	
Mild in 2009									
Nondrinker in 2011	253 643	723	0.5	1.94 (1.76-2.14)	<.001	1.35 (1.21-1.50)	<.001	1.33 (1.20-1.48)	<.001
Mild in 2011	625 723	922	0.2	1 [Reference]		1 [Reference]		1 [Reference]	
Moderate in 2011	130 116	219	0.3	1.13 (0.97-1.31)		1.08 (0.93-1.26)		1.06 (0.92-1.23)	
Heavy in 2011	39 096	120	0.5	2.07 (1.70-2.51)		1.54 (1.27-1.88)		1.48 (1.22-1.79)	
Moderate in 2009									
Nondrinker in 2011	36 329	140	0.6	2.18 (1.78-2.68)	<.001	1.22 (0.98-1.51)	.003	1.27 (1.03-1.57)	.003
Mild in 2011	151 249	260	0.3	0.99 (0.84-1.17)		0.83 (0.70-0.98)		0.84 (0.71-0.99)	
Moderate in 2011	168 501	295	0.3	1 [Reference]		1 [Reference]		1 [Reference]	
Heavy in 2011	75 124	159	0.3	1.19 (0.98-1.44)		1.03 (0.84-1.25)		1.03 (0.85-1.25)	
Heavy in 2009									
Nondrinker in 2011	22 604	109	0.8	1.99 (1.59-2.48)	<.001	1.35 (1.07-1.70)	.08	1.39 (1.11-1.74)	.05
Mild in 2011	49 657	135	0.4	1.17 (0.96-1.43)		1.07 (0.88-1.31)		1.05 (0.86-1.28)	
Moderate in 2011	83 299	202	0.4	1.01 (0.85-1.21)		1.10 (0.92-1.31)		1.10 (0.92-1.31)	
Heavy in 2011	140 915	344	0.4	1 [Reference]		1 [Reference]		1 [Reference]	

Abbreviations: HR, hazard ratio; IR, incidence rate; PY, person-year.

^a^ Model 1 was adjusted for age, sex, smoking status, physical activity, area of residence, and income.

^b^ Model 2 included model 1 plus body mass index, hypertension, diabetes, dyslipidemia, systolic blood pressure, fasting glucose level, total cholesterol level, and serum creatinine.

**Figure.**
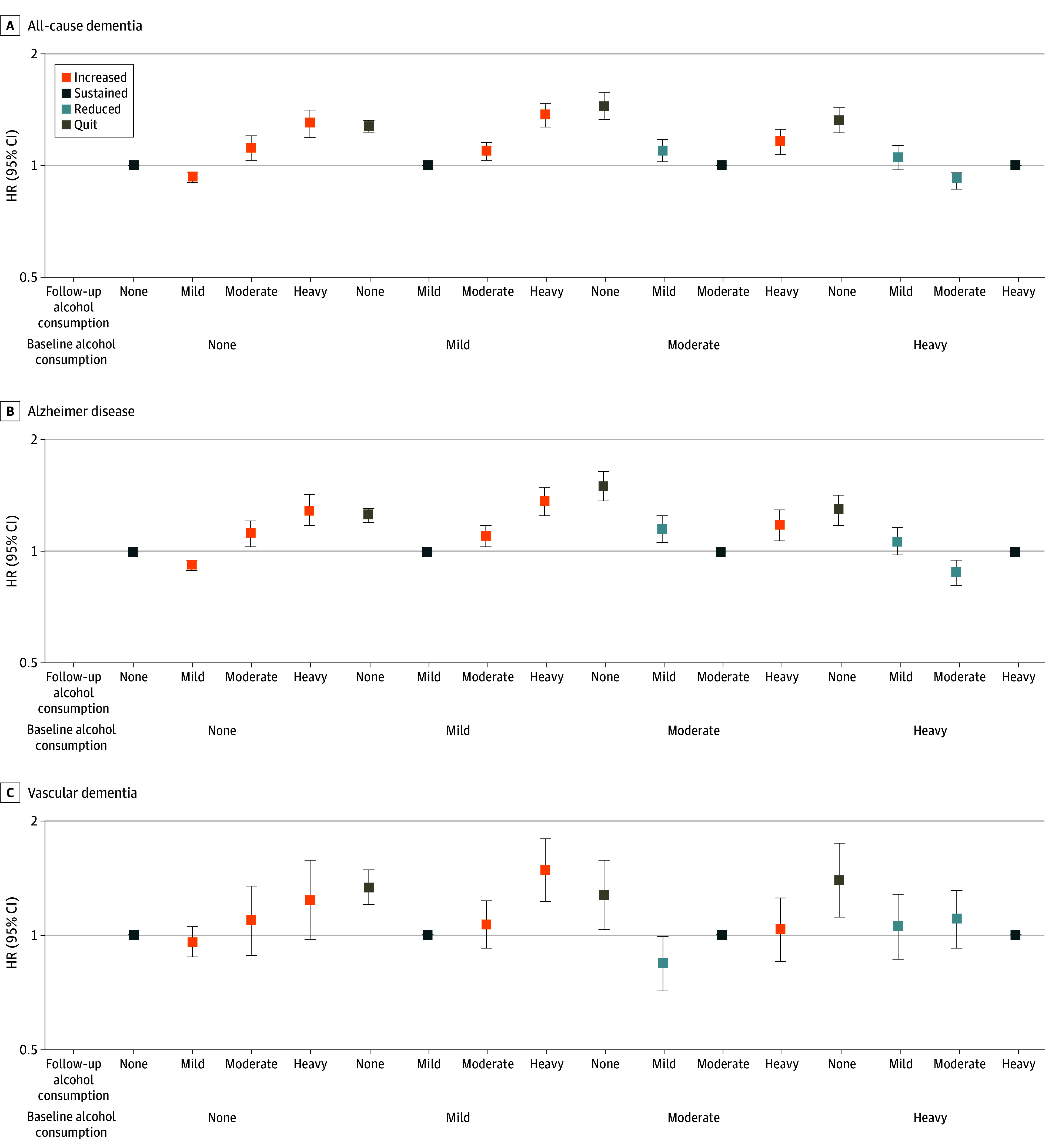
Adjusted Hazard Ratios (HRs) and 95% CIs for the Association of Change in Alcohol Consumption Amount With Risk of Dementia HRs (squares) were adjusted for age, sex, smoking status, regular exercise, area of residence, income, comorbidities (hypertension, diabetes, and dyslipidemia), systolic blood pressure, and laboratory results (fasting glucose levels, total cholesterol, and serum creatinine). Error bars denote 95% CIs.

### Subgroup Analysis: Subsequent Changes in Alcohol Consumption and Risk of Dementia

When we extended the analysis using additional data on alcohol consumption in the subgroup who underwent a health examination in 2013, the results demonstrated similar patterns (eTables 10 in Supplement 1). Those who sustained a mild-moderate level of drinking through the third examination (aHR, 0.75; 95% CI, 0.72-0.77) and those who initiated a mild-moderate level of drinking at the 2011 screening and maintained a mild-moderate level (aHR, 0.82; 95% CI, 0.77-0.88) were found to have a lower risk of all-cause dementia compared with sustained nondrinkers. Among those who reduced their alcohol intake from a heavy level in 2009 to a mild-moderate level in 2011 and mild-moderate level in 2013, there was a decreased aHR for all-cause dementia (aHR, 0.92, 95% CI, 0.83-1.03), although the difference was not significant. In contrast, those who quit at the 2011 screening and sustained quitting were at a higher risk of all-cause dementia and AD.

## Discussion

In this nationwide cohort study, we found that sustained mild drinkers had a 21% decreased risk of all-cause dementia, and sustained moderate drinkers had a 17% decreased risk of all-cause dementia compared with sustained nondrinkers, whereas sustained heavy drinkers had an 8% increased risk. Compared with those who sustained the same level of drinking, heavy drinkers who reduced intake to a moderate level and nondrinkers who initiated drinking to a mild level exhibited a decreased risk of all-cause dementia and AD, whereas those who increased alcohol consumption from a mild or a moderate level to a heavy level exhibited an increased risk of all-cause dementia and AD. Subgroup analysis using information from a third examination showed consistent findings regarding a subsequent change in drinking level, supporting the robustness of our results.

We observed a J-shaped or U-shaped association between alcohol consumption and risk of all-cause dementia, which is consistent with the majority of previous studies.^18^ We also found a similar pattern of associations between alcohol consumption and risk of AD and VaD. Consistent with our findings, in a recent systematic review of meta-analyses,^19^ mild to moderate alcohol consumption was found to be protective for all-cause dementia, AD, and VaD. The protective effect of mild to moderate alcohol consumption may be attributed to various mechanisms. In AD, for example, previous studies^20^ proposed that the protective effect of mild to moderate alcohol consumption involves the promotion of prosurvival pathways and decrease of neuroinflammation. Regarding VaD, previous studies^21,22^ proposed that mild to moderate alcohol consumption may be beneficial to the vascular system, with beneficial effects on platelet function and increased serum concentration of high-density lipoprotein. However, excessive alcohol consumption also has various harmful effects through direct mechanisms, such as the proven neurotoxic effect of alcohol and nutritional deficiency.^23^ Additionally, excessive alcohol consumption is thought to exacerbate the pathology of AD through enhanced tau accumulation^24^ and the destruction of cholinergic neurons accompanied by decreased acetylcholine release.^25^

Our study showed that initiation of mild alcohol consumption was associated with a decreased risk of all-cause dementia and AD, which, to our knowledge, has never been reported in previous studies. Although mild to moderate alcohol consumption has been reported to confer beneficial effects on cardiovascular disease,^26,27^ debate persists with respect to numerous other outcomes.^28^ None of the existing health guidelines recommends starting alcohol drinking. The 2015 to 2020 Dietary Guidelines for Americans does not recommend that individuals begin drinking or drink more for any reason.^13^ Moreover, personal metabolic characteristics (sex, body weight, and acetaldehyde dehydrogenase type^29^) and susceptibility to alcohol vary individually, thereby making it difficult to find the optimal level of alcohol for each individual. Furthermore, alcohol consumption is a marker for several lifestyle factors, and a mild to moderate level of drinking is considered an important component of social activities. Several studies suggest that more frequent social contact decreases the risk of dementia.^30^ It is difficult to draw conclusions from our results without fully understanding the socioeconomic reasons underlying the changes in drinking patterns. Given the ethical limitations of a randomized clinical trial able to sufficiently establish causality, additional studies that further support our conclusion are required before clinical application of these findings.

Our results showed that quitting from any level of alcohol consumption was associated with a higher risk of all-cause dementia, AD, and VaD, which is in line with a previous report.^4,5^ The results observed in the quitters are suspected to be primarily attributed to the sick quitter effect, which is defined as a person quitting (or reducing) a certain hazardous activity because of health issues.^31^ Sabia et al^4^ found the excess risk of dementia associated with abstinence was partly explained by cardiometabolic disease (stroke, coronary heart disease, atrial fibrillation, heart failure, and diabetes). Uncaptured medical comorbidities or health consequences leading to quitting may exist in our study. To minimize possible reverse causality, we conducted a subgroup analysis with 3 assessments and applied a 1-year lag time, but the sick quitter effect remains a source of potential bias.

In our study, nondrinkers seemed to have other risk factors, including being older and lower income. Although existing evidence points to a downward trend in alcohol consumption as people age,^32,33^ in our age-stratified analyses, the association between change in alcohol consumption with risk of dementia was similar between the younger than 65 years age group and the 65 years and older age group. Regarding the association of socioeconomic status with alcohol consumption, people in higher socioeconomic status groups seem to drink more often and drink smaller amounts more frequently, whereas lower socioeconomic status groups have a higher proportion of abstainers, but otherwise drink more often in problematic ways.^34^ Although we adjusted for several socioeconomic characteristics in our analyses, we admit that the measures did not fully address the social capital aspect of socioeconomic status and health problems. Thus, the exact nature of these complex relationships warrants further investigation.

Interestingly, reducing alcohol consumption from a heavy (≥30 g per day) to a moderate level (15-29.9 g per day) was associated with an 8% decreased risk of all-cause dementia and a 12% decreased risk of AD in our study. In subgroup analysis of participants who underwent 3 health examinations, in which mild and moderate drinking levels were merged into a single mild-moderate level because of the small number of cases, among those who reduced their alcohol intake from a heavy level in 2009 to mild-moderate level in 2011 and mild-moderate level in 2013, there was a decreased aHR for all-cause dementia (aHR, 0.92; 95% CI, 0.83-1.03). A possible interpretation of this result is that sustaining heavy alcohol drinking is detrimental to dementia, which may be conceptually in line with a recent indication that excessive alcohol consumption (drinking >21 units of alcohol per week = 24 g per day) has been recognized as a new modifiable risk factor for dementia in the 2020 dementia prevention guidelines published by the Lancet Commission.^30^

### Limitations

This study has several limitations. First, alcohol consumption was self-reported in our study, which tends to underestimate the actual level of alcohol consumption.^35^ Second, the type of alcoholic beverage was not considered in our study.^36,37^ Third, our study participants were limited to health screening participants, who might be healthier and more engaged in a healthy lifestyle than the general population.^38^ Fourth, although our models were adjusted for various potential confounders, unmeasured confounders, including genetic ones (eg, *APOE*),^5,9^ might still distort the results. Fifth, caution is required when applying our results to ethnic groups other than Korean individuals, because the genetic background for alcohol metabolism^39^ and drinking culture vary depending on ethnicity.

## Conclusions

In conclusion, our analyses indicate that maintaining mild to moderate alcohol consumption is associated with a decreased risk of dementia, whereas maintaining heavy drinking is associated with an increased risk of dementia. Notably, our analyses stratified by the initial amount of alcohol consumption indicate that reduction of drinking from a heavy to a moderate level and initiation of mild drinking were associated with a decreased risk of all-cause dementia and AD.

## Notes

Supplement 1. **eMethods.** Information on Covariates in the Study
eReferences
**eTable 1.** Previous Studies on the Relationship Between Change in Alcohol Consumption and the Incidence of Dementia **eTable 2. **Change in Drinking Level Between 2009 and 2011 **eTable 3.** Change in Drinking Patterns Between 2009 and 2011 **eTable 4.** Baseline Characteristics of the Study Population According to Sex **eTable 5.** Subdistribution Hazard Ratios (SHR) and 95% Confidence Intervals (CI) for the Association Between Change in Alcohol Consumption and the Risk of Dementia: Sensitivity Analysis Accounting for the Competing Risk of Death **eTable 6.** Hazard Ratios (HR) and 95% Confidence Intervals (CI) for the Association Between Change in Alcohol Consumption Amount and the Risk of Dementia Among Males **eTable 7.** Hazard Ratios (HR) and 95% Confidence Intervals (CI) for the Association Between Change in Alcohol Consumption Amount and the Risk of Dementia Among Females **eTable 8.** Hazard Ratios and 95% Confidence Intervals for Dementia According to Change in Drinking Level by Age **eTable 9.** Hazard Ratios and 95% Confidence Intervals for Dementia According to Change in Drinking Level by Smoking **eTable 10.** Associations Between Changes in Drinking Level From 2009 to 2013 and Dementia **eFigure 1.** Flow Chart of Study Population **eFigure 2.** Directed Acyclic Graph (DAG) Illustrating the Assumptions About the Causal Relationship Between Changes in Alcohol Consumption and Dementia

Supplement 2.
Data Sharing Statement
