# Supplementary material for: Changes in Alcohol Consumption and Risk of Dementia in a Nationwide Cohort in South Korea
Source: JAMA Netw Open. 2023 Feb 6;6(2):e2254771. doi: 10.1001/jamanetworkopen.2022.54771 (PMC12549098; doi:10.1001/jamanetworkopen.2022.54771)
Supplement: Supplement 1. — eMethods. Information on Covariates in the Study eReferences eTable 1. Previous Studies on the Relationship Between Change in Alcohol Consumption and the Incidence of Dementia eTable 2. Change in Drinking Level Between 2009 and 2011 eTable 3. Change in Drinking Patterns Between 2009 and 2011 eTable 4. Baseline Characteristics of the Study Population According to Sex eTable 5. Subdistribution Hazard Ratios (SHR) and 95% Confidence Intervals (CI) for the Association Between Change in Alcohol Consumption and the Risk of Dementia: Sensitivity Analysis Accounting for the Competing Risk of Death eTable 6. Hazard Ratios (HR) and 95% Confidence Intervals (CI) for the Association Between Change in Alcohol Consumption Amount and the Risk of Dementia Among Males eTable 7. Hazard Ratios (HR) and 95% Confidence Intervals (CI) for the Association Between Change in Alcohol Consumption Amount and the Risk of Dementia Among Females eTable 8. Hazard Ratios and 95% Confidence Intervals for Dementia According to Change in Drinking Level by Age eTable 9. Hazard Ratios and 95% Confidence Intervals for Dementia According to Change in Drinking Level by Smoking eTable 10. Associations Between Changes in Drinking Level From 2009 to 2013 and Dementia eFigure 1. Flow Chart of Study Population eFigure 2. Directed Acyclic Graph (DAG) Illustrating the Assumptions About the Causal Relationship Between Changes in Alcohol Consumption and Dementia [file jamanetwopen-e2254771-s001.pdf]

Supplemental Online Content

Jeon KH, Han K, Jeong SM, et al. Changes in alcohol consumption and risk of dementia in a nationwide cohort in South Korea. *JAMA Netw Open*. 2023;6(2):e2254771. doi:10.1001/jamanetworkopen.2022.54771

eMethods. Information on Covariates in the Study

eReferences

eTable 1. Previous Studies on the Relationship Between Change in Alcohol Consumption and the Incidence of Dementia

eTable 2. Change in Drinking Level Between 2009 and 2011

eTable 3. Change in Drinking Patterns Between 2009 and 2011

eTable 4. Baseline Characteristics of the Study Population According to Sex

eTable 5. Subdistribution Hazard Ratios (SHR) and 95% Confidence Intervals (CI) for the Association Between Change in Alcohol Consumption and the Risk of Dementia: Sensitivity Analysis Accounting for the Competing Risk of Death

eTable 6. Hazard Ratios (HR) and 95% Confidence Intervals (CI) for the Association Between Change in Alcohol Consumption Amount and the Risk of Dementia Among Males

eTable 7. Hazard Ratios (HR) and 95% Confidence Intervals (CI) for the Association Between Change in Alcohol Consumption Amount and the Risk of Dementia Among Females

eTable 8. Hazard Ratios and 95% Confidence Intervals for Dementia According to Change in Drinking Level by Age

eTable 9. Hazard Ratios and 95% Confidence Intervals for Dementia According to Change in Drinking Level by Smoking

eTable 10. Associations Between Changes in Drinking Level From 2009 to 2013 and Dementia

eFigure 1. Flow Chart of Study Population

eFigure 2. Directed Acyclic Graph (DAG) Illustrating the Assumptions About the Causal Relationship Between Changes in Alcohol Consumption and Dementia

This supplemental material has been provided by the authors to give readers additional information about their work.

© 2023 Jeon KH et al. *JAMA Network Open.*

eMethods. Information on Covariates in the Study

Information on all covariates was based on results from the second health exam in 2011. Smoking status was categorized as never smoker, ex-smoker, or current smoker based on questionnaire responses. Regular exercise was defined as performing at least 150 min of moderate-intensity or 75 min of vigorous-intensity physical activity per week.[1,2] Body mass index (BMI) was calculated as weight (kg) divided by the square of height in meters (m^2). Comorbidities were defined based on medical claims according to the 10th edition of the International Classification of Diseases (ICD-10) codes (for hypertension, I10-I13 or I15; for diabetes, E11–E14; for dyslipidemia, E78; and for chronic kidney disease, N18 or N19). Income was categorized into quartiles based on insurance premium levels (in Korea, insurance premiums are determined by income level), with those covered by Medical Aid (the poorest 3%) merged into the lowest income quartile.

eReferences

1. Arnett DK, Blumenthal RS, Albert MA, et al. 2019 ACC/AHA Guideline on the Primary Prevention of Cardiovascular Disease: A Report of the American College of Cardiology/American Heart Association Task Force on Clinical Practice Guidelines. Circulation. 2019;140(11):e596-e646.

2. Bull FC, Al-Ansari SS, Biddle S, et al. World Health Organization 2020 guidelines on physical activity and sedentary behaviour. British journal of sports medicine. 2020;54(24):1451-1462.

© 2023 Jeon KH et al. *JAMA Network Open.*

eTable 1. Previous Studies on the Relationship Between Change in Alcohol Consumption and the Incidence of Dementia

Study	Study design /country	No. of participants	Age range (years)	Follow-up (years)	Adjustment	Outcomes	No. of outcomes	Alcohol assessment	Group	Result
Sabia et al. (2018)	Prospective cohort study (Whitehall II study) /UK	8927	35–55	12.9	Age, sex, ethnicity, education, occupation, marital status, smoking, exercise, fruit and vegetable consumption, SBP, TC, BMI, diabetes, CVD, CVD drugs, general health questionnaire score	Dementia (ICD-10 codes F00-F03, F05.1, G30, and G31)	396	Trajectories of alcohol consumption between 1985/88 and 2002/04 (at least two assessments in 1985/88, 1989/90, 1991/93, 1997/99, and 2002/04)	 Long-term abstinence<br>Decreased alcohol consumption<br>Long-term consumption of 1–14 units/week<br>Increased consumption<br>Long-term consumption of >14 units/week	[HR (95%CI)]<br>**1.67 (1.26–2.23)**<br>**1.50 (1.04–2.16)**<br>1.00 (ref)<br>0.85 (0.57–1.26)<br>1.36 (0.99–1.88)
Mukamal et al. (2003)	Nested case-control study (Cardiovascular Health Study) /US	746	≥ 65	6	Age, sex, ethnicity, education, income, physical activity, smoking, diabetes, TC, BMI, use of HRT, APOE4 status, atrial fibrillation, history of CHF, stroke, and TIA	DSM-IV criteria	373	Averaged alcohol consumption from the baseline questionnaire and the questionnaire from the annual clinic visit closest to date of the MRI examination	 Abstainers†<br>Quitters (those who quit between the baseline examination and the beginning of follow-up)	[OR (95%CI)]<br>1.00 (ref)<br>All-cause dementia<br>1.38 (0.81–2.35)<br>Alzheimer disease<br>1.18 (0.68–2.04)<br>Vascular dementia<br>1.39 (0.63–3.08)

HR, hazard ratio; OR, odds ratio; SBP, systolic blood pressure; TC, total cholesterol; BMI, body mass index; HRT, hormone replacement therapy; CVD, cardiovascular disease; CHF, congestive failure; TIA, transient ischemic attack

Statistically significant values are marked in bold.

†Abstainers without former use (those who reported prior alcohol consumption at the baseline examination) as the reference category

© 2023 Jeon KH et al. *JAMA Network Open.*

eTable 2. Change in Drinking Level Between 2009 and 2011

2009 \ 2011	Non-drinker	Mild drinker	Moderate drinker	Heavy drinker	Total
Non-drinker	1,857,920	243,958	34,821	20,427	2,157,126 (54.8)
Mild drinker	253,643	625,723	130,116	39,096	1,048,578 (26.7)
Moderate drinker	36,329	151,249	168,501	75,124	431,203 (11.0)
Heavy drinker	22,604	49,657	83,299	140,915	296,475 (7.5)
Total	2,170,496 (55.2)	1,070,587 (27.2)	416,737 (10.6)	275,562 (7.0)	3,933,382 (100)

Data are expressed as number (%)

□ non-drinker or sustainer; ■ quitter; ■ reducer; ■ increaser

© 2023 Jeon KH et al. *JAMA Network Open.*

eTable 3. Change in Drinking Patterns Between 2009 and 2011

	**Participant drinking level, No, (%)**									
	**Non-drinkers (N = 1,857,920)**		**Sustainers (N = 935,139)**		**Quitters (N = 312,576)**		**Reducers (N = 284,205)**		**Increasers (N = 543,542)**	
	**2009**	**2011**	**2009**	**2011**	**2009**	**2011**	**2009**	**2011**	**2009**	**2011**
**Daily alcohol intake**										
Non-drinker	1,857,920 (100)	1,857,920 (100)	NA	NA	NA	312,576 (100)	NA	NA	299,206 (55.1)	NA
Mild (< 15g/day)	NA	NA	625,723 (66.9)	625,723 (66.9)	253,643 (81.1)	NA	NA	Moderate → Mild 151,249 (53.2)<br>Heavy → Mild 49,657 (17.5)	169,212 (31.1)	None → Mild 243,958 (44.9)
Moderate (15–29 g/day)	NA	NA	168,501 (18.0)	168,501 (18.0)	36,329 (11.6)	NA	151,249 (53.2)	Heavy → Moderate 83,299 (29.3)	75,124 (13.8)	None → Moderate 34,821 (6.4)<br>Mild → Moderate 130,116 (23.9)
Heavy (≥ 30 g/day)	NA	NA	140,915 (15.1)	140,915 (15.1)	22,604 (7.2)	NA	132,956 (46.8)	NA	NA	None → Heavy 20,427 (3.8)<br>Mild → Heavy 39,096 (7.2)<br>Moderate → Heavy 75,124 (13.8)
**Frequency (days per week)**										
None	1,857,920 (100)	1,857,920 (100)	NA	NA	NA	312,576 (100)	NA	NA	299,206 (55.1)	NA
1–2	NA	NA	671,877 (71.9)	657,878 (70.4)	257,672 (82.4)	NA	108,653 (38.2)	190,966 (67.2)	172,549 (31.8)	333,055 (61.3)
3–4	NA	NA	179,840 (19.2)	188,106 (20.1)	34,465 (11.0)	NA	116,810 (41.1)	76,329 (26.9)	59,545 (11.0)	137,456 (25.3)
5–7	NA	NA	83,422 (8.9)	89,155 (9.5)	20,439 (6.5)	NA	58,742 (20.7)	16,910 (6.0)	12,242 (2.3)	73,031 (13.4)
**Amount (drinks per occasion)**										
None	1,857,920 (100)	1,857,920 (100)	NA	NA	NA	312,576 (100)	NA	NA	299,206 (55.1)	NA
1–2	NA	NA	143,332 (15.3)	175,094 (18.7)	111,188 (35.6)	NA	2,330 (0.8)	36,476 (12.8)	23,614 (4.3)	120,252 (22.1)
3–4	NA	NA	258,408 (27.6)	258,421 (27.6)	94,959 (30.4)	NA	15,979 (5.6)	79,555 (28.0)	60,192 (11.1)	101,387 (18.7)
5–7	NA	NA	318,239 (34.0)	308,132 (33.0)	71,244 (22.8)	NA	121,141 (42.6)	117,505 (41.4)	106,239 (19.6)	176,730 (32.5)

© 2023 Jeon KH et al. *JAMA Network Open.*

	Participant drinking level, No, (%)									
	**Non-drinkers (N = 1,857,920)**		**Sustainers (N = 935,139)**		**Quitters (N = 312,576)**		**Reducers (N = 284,205)**		**Increasers (N = 543,542)**	
≥ 8	NA	NA	215,160 (23.0)	193,492 (20.7)	35,185 (11.3)	NA	144,755 (50.9)	50,669 (17.8)	54,291 (10.0)	145,173 (26.7)

Abbreviation: NA, non-applicable

© 2023 Jeon KH et al. *JAMA Network Open.*

eTable 4. Baseline Characteristics of the Study Population According to Sex

	Change in daily amount of alcohol consumption between 2009 and 2011				
**Variable**	Non-drinkers	Quitters	Reducers	Sustainers	Increasers
Male					
Number	477,073	172,112	258,738	741,691	388,334
Age, mean (SD), y	57.7 (10.3)	55.9 (10.0)	53.0 (8.7)	52.8 (8.6)	53.5 (9.0)
Alcohol consumption status in 2009, No. (%)					
Non-drinker	477,073 (100)	NA	NA	NA	165,084 (42.5)
Mild (< 15g/day)	NA	123,471 (71.7)	NA	444,476 (59.9)	150,995 (38.9)
Moderate (15–29 g/day)	NA	28,663 (16.7)	133,392 (51.6)	159,435 (21.5)	72,255 (18.6)
Heavy (≥ 30 g/day)	NA	19,978 (11.6)	125,346 (48.4)	137,780 (18.6)	NA
Smoking status, No. (%)					
None	215,795 (45.2)	83,948 (48.8)	53,695 (20.8)	171,768 (23.2)	93,953 (24.2)
Former smoker	139,889 (29.3)	45,707 (26.6)	88,504 (34.2)	271,917 (36.7)	135,886 (35.0)
Current smoker					
< 10 cigarettes/day	11,376 (2.4)	5,073 (3.0)	11,311 (4.4)	29,002 (3.9)	14,473 (3.7)
10–19 cigarettes/day	44,090 (9.2)	16,299 (9.5)	46,485 (18.0)	119,394 (16.1)	57,700 (14.9)
≥ 20 cigarettes/day	65,923 (13.8)	21,085 (12.3)	58,743 (22.7)	149,610 (20.2)	86,322 (22.2)
Regular physical activity, No. (%)					
None	362,693 (76.0)	134,874 (78.4)	193,790 (74.9)	554,229 (74.7)	291,369 (75.0)
Yes	114,380 (24.0)	37,238 (21.6)	64,948 (25.1)	187,462 (25.3)	96,965 (25.0)
Anthropometrics, mean (SD)					
Body mass index, kg/m^2	24 (2.9)	24.1 (2.9)	24.4 (2.8)	24.2 (2.8)	24.3 (2.8)
Waist circumference, cm	83.8 (7.7)	84.1 (7.5)	84.7 (7.3)	84.2 (7.2)	84.4 (7.4)
Systolic blood pressure, mmHg	124.2 (14.2)	124.8 (14.2)	126.9 (14.1)	125.9 (14.0)	126.3 (14.3)
Diastolic blood pressure, mmHg	77.1 (9.5)	77.8 (9.6)	79.7 (9.7)	79.1 (9.6)	79.2 (9.7)
Comorbidity					
Hypertension, No. (%)	162,851 (34.1)	62,531 (36.3)	98,497 (38.1)	257,645 (34.7)	141,539 (36.5)
Diabetes mellitus, No. (%)	67,056 (14.1)	25,296 (14.7)	35,724 (13.8)	89,723 (12.1)	52,530 (13.5)
Dyslipidemia, No. (%)	95,972 (20.1)	35,940 (20.9)	54,404 (21.0)	148,189 (20.0)	77,869 (20.1)
Chronic kidney disease, No. (%)	30,299 (6.4)	9,205 (5.4)	10,260 (4.0)	30,551 (4.1)	16,067 (4.1)
Laboratory findings, mean (SD)					
Glucose, mg/dL	100.9 (25.8)	102.1 (26.7)	103.6 (26.4)	102.2 (25.0)	103.2 (26.7)
Total cholesterol, mg/dL	193.5 (35.9)	194.4 (36.1)	197.2 (35.6)	197.4 (35.1)	196.9 (35.5)
HDL, mg/dL	49.3 (15.1)	50.8 (15.8)	53.9 (17.0)	53.6 (17.0)	53.8 (17.5)
LDL, mg/dL	117.6 (33.3)	115.6 (33.8)	112.0 (34.8)	114.0 (34.1)	113.0 (34.4)
GFR, mL/min/1.73 m^2	85.9 (37.0)	87.2 (36.1)	88.7 (38.9)	88.0 (39.9)	88.3 (37.2)
Urban residency, No. (%)	206,953 (43.4)	75,726 (44.0)	117,596 (45.5)	349,160 (47.1)	174,177 (44.9)

© 2023 Jeon KH et al. *JAMA Network Open.*

	Change in daily amount of alcohol consumption between 2009 and 2011				
**Variable**	Non-drinkers	Quitters	Reducers	Sustainers	Increasers
Income level by quartile, No. (%)					
Q1 (lowest)	87,281 (18.9)	27,261 (16.4)	35,286 (14.1)	92,793 (12.9)	55,787 (14.8)
Q2	83,409 (18.0)	27,794 (16.7)	40,277 (16.1)	104,596 (14.6)	59,658 (15.9)
Q3	121,325 (26.2)	42,785 (25.8)	66,343 (26.5)	180,903 (25.2)	96,726 (25.7)
Q4 (highest)	170,388 (36.9)	68,169 (41.1)	108,153 (43.3)	339,235 (47.3)	163,720 (43.6)
Female					
Number	1,380,847	140,464	25,467	193,448	155,208
Age, mean (SD), y	57.2 (9.8)	53.5 (8.9)	50.8 (7.0)	50.1 (7.1)	52.0 (8.4)
Alcohol consumption status in 2009, No. (%)					
Non-drinker	1,380,847 (100)	NA	NA	NA	134,122 (86.4)
Mild (< 15g/day)	NA	130,172 (92.7)	NA	181,247 (93.7)	18,217 (11.7)
Moderate (15–29 g/day)	NA	7,666 (5.5)	17,857 (70.1)	9,066 (4.7)	2,869 (1.8)
Heavy (≥ 30 g/day)	NA	2,626 (1.9)	7,610 (29.9)	3,135 (1.6)	NA
Smoking status, No. (%)					
None	1,356,788 (98.3)	135,447 (96.4)	20,643 (81.1)	178,983 (92.5)	144,340 (93.0)
Former smoker	8,322 (0.6)	1,621 (1.2)	1,295 (5.1)	4,917 (2.5)	3,356 (2.2)
Current smoker					
< 10 cigarettes/day	6,029 (0.4)	1,431 (1.0)	1,318 (5.2)	3,991 (2.1)	2,852 (1.8)
10–19 cigarettes/day	6,760 (0.5)	1,386 (1.0)	1,556 (6.1)	4,109 (2.1)	3,175 (2.1)
≥ 20 cigarettes/day	2,948 (0.2)	579 (0.4)	655 (2.6)	1,448 (0.8)	1,485 (1.0)
Regular physical activity, No. (%)					
None	1,119,223 (81.1)	113,806 (81.0)	19,889 (78.1)	151,212 (78.2)	121,624 (78.4)
Yes	261,624 (18.9)	26,658 (19.0)	5,578 (21.9)	42,236 (21.8)	33,584 (21.6)
Anthropometrics, mean (SD)					
Body mass index, kg/m^2	23.8 (3.1)	23.8 (3.1)	23.9 (3.1)	23.4 (3.0)	23.6 (3.0)
Waist circumference, cm	78.1 (8.3)	77.4 (8.2)	78.0 (8.3)	76.3 (7.9)	77.1 (8.2)
Systolic blood pressure, mmHg	122.4 (15.5)	120.8 (15.1)	121.7 (14.9)	119.6 (14.5)	120.5 (14.9)
Diastolic blood pressure, mmHg	75.3 (9.9)	75.1 (9.9)	76.4 (10.1)	74.9 (9.9)	75.2 (9.9)
Comorbidity					
Hypertension, No. (%)	474,495 (34.4)	39,761 (28.3)	7,331 (28.8)	43,419 (22.4)	40,031 (25.8)
Diabetes mellitus, No. (%)	136,961 (9.9)	9,949 (7.1)	1,605 (6.3)	8,744 (4.5)	9,508 (6.1)
Dyslipidemia, No. (%)	389,139 (28.2)	34,466 (24.5)	5,644 (22.2)	37,257 (19.3)	33,586 (21.6)
Chronic kidney disease, No. (%)	97,946 (7.1)	7,049 (5.0)	889 (3.5)	7,288 (3.8)	6,903 (4.5)
Laboratory findings, mean (SD)					
Glucose, mg/dL	97.0 (20.9)	96.0 (19.3)	97.1 (18.6)	94.8 (17.1)	95.8 (18.9)
Total cholesterol, mg/dL	201.9 (37.1)	201.7 (36.9)	201.8 (36.3)	199.9 (35.3)	200.9 (36.0)
HDL, mg/dL	56.6 (16.7)	59.0 (18.1)	63.3 (19.2)	62.1 (17.2)	61.0 (19.2)

© 2023 Jeon KH et al. *JAMA Network Open.*

	Change in daily amount of alcohol consumption between 2009 and 2011				
**Variable**	Non-drinkers	Quitters	Reducers	Sustainers	Increasers
LDL, mg/dL	121.5 (34.1)	120.3 (33.7)	115.0 (33.8)	116.7 (32.5)	118.0 (33.0)
GFR, mL/min/1.73 m^2	87.0 (28.5)	88.5 (27.5)	90.6 (27.0)	89.4 (26.4)	89.4 (29.3)
Urban residency, No. (%)	611,410 (44.3)	65,098 (46.3)	12,173 (47.8)	94,978 (49.1)	71,593 (46.1)
Income level by quartile, No. (%)					
Q1 (lowest)	342,654 (25.6)	38,882 (28.4)	7,412 (29.6)	54,610 (28.8)	44,597 (29.4)
Q2	268,048 (20.0)	32,479 (23.7)	7,006 (28.0)	49,024 (25.8)	36,649 (24.2)
Q3	303,170 (22.6)	30,275 (22.1)	5,606 (22.4)	39,711 (20.9)	32,276 (21.3)
Q4 (highest)	425,351 (31.8)	35,334 (25.8)	4,984 (19.9)	46,365 (24.4)	38,179 (25.2)

Abbreviation: NA, non-applicable

© 2023 Jeon KH et al. *JAMA Network Open.*

eTable 5. Subdistribution Hazard Ratios (SHR) and 95% Confidence Intervals (CI) for the Association Between Change in Alcohol Consumption and the Risk of Dementia: Sensitivity Analysis Accounting for the Competing Risk of Death

Alcohol consumption status		Subjects (N)	Events (N)	IR (per 1000 PYs)	**Non-drinkers as a reference**			**Sustained drinking at the same level as a reference**	
2009	2011				aHR[a] (95% CI)	aSHR[a] (95% CI)		aHR[a] (95% CI)	aSHR[a] (95% CI)
**All-cause dementia**									
Non-drinker	Non	1,857,920	68,679	5.9	1 (Ref.)	1 (Ref.)		1 (Ref.)	1 (Ref.)
	Mild	243,958	4,338	2.8	0.93 (0.90–0.96)	0.93 (0.90–0.96)		0.93 (0.90–0.96)	0.93 (0.90–0.96)
	Moderate	34,821	718	3.3	1.11 (1.03–1.20)	1.12 (1.04–1.21)		1.11 (1.03–1.20)	1.12 (1.04–1.21)
	Heavy	20,427	556	4.4	1.30 (1.19–1.41)	1.29 (1.18–1.41)		1.30 (1.19–1.41)	1.29 (1.18–1.41)
Mild	Non	253,643	6,153	3.9	1.02 (1.00–1.05)	1.02 (0.99–1.05)		1.27 (1.23–1.32)	1.27 (1.22–1.32)
	Mild	625,723	6,690	1.7	0.79 (0.77–0.81)	0.80 (0.77–0.82)		1 (Ref.)	1 (Ref.)
	Moderate	130,116	1,471	1.8	0.86 (0.82–0.91)	0.86 (0.82–0.91)		1.09 (1.03–1.15)	1.08 (1.02–1.15)
	Heavy	39,096	767	3.1	1.10 (1.02–1.18)	1.11 (1.03–1.20)		1.37 (1.27–1.47)	1.38 (1.27–1.49)
Moderate	Non	36,329	1,060	4.7	1.23 (1.16–1.31)	1.21 (1.13–1.29)		1.44 (1.33–1.57)	1.40 (1.29–1.53)
	Mild	151,249	1,929	2.0	0.92 (0.88–0.96)	0.92 (0.88–0.97)		1.09 (1.02–1.17)	1.09 (1.02–1.17)
	Moderate	168,501	1,521	1.4	0.83 (0.79–0.88)	0.84 (0.80–0.88)		1 (Ref.)	1 (Ref.)
	Heavy	75,124	986	2.1	0.98 (0.92–1.04)	0.98 (0.92–1.05)		1.16 (1.07–1.25)	1.15 (1.06–1.25)
Heavy	Non	22,604	883	6.3	1.42 (1.33–1.52)	1.38 (1.29–1.48)		1.32 (1.22–1.43)	1.28 (1.18–1.40)
	Mild	49,657	1,005	3.2	1.12 (1.06–1.20)	1.12 (1.05–1.20)		1.05 (0.97–1.13)	1.04 (0.96–1.13)
	Moderate	83,299	1,153	2.2	0.98 (0.93–1.05)	0.98 (0.92–1.04)		0.92 (0.86–0.99)	0.91 (0.85–0.98)
	Heavy	140,915	2,373	2.7	1.08 (1.03–1.12)	1.08 (1.03–1.13)		1 (Ref.)	1 (Ref.)
Alzheimer's disease									
Non-drinker	Non	1,857,920	55,823	4.8	1 (Ref.)	1 (Ref.)		1 (Ref.)	1 (Ref.)
	Mild	243,958	3,416	2.2	0.93 (0.90–0.96)	0.94 (0.90–0.97)		0.92 (0.89–0.95)	0.93 (0.90–0.96)
	Moderate	34,821	559	2.6	1.13 (1.04–1.23)	1.14 (1.04–1.24)		1.12 (1.03–1.21)	1.13 (1.03–1.23)
	Heavy	20,427	427	3.4	1.30 (1.18–1.43)	1.28 (1.15–1.41)		1.29 (1.18–1.43)	1.27 (1.15–1.41)
Mild	Non	253,643	4,817	3.0	1.01 (0.98–1.04)	1.00 (0.97–1.04)		1.26 (1.20–1.31)	1.24 (1.19–1.30)
	Mild	625,723	5,066	1.3	0.78 (0.75–0.80)	0.78 (0.76–0.81)		1 (Ref.)	1 (Ref.)
	Moderate	130,116	1,104	1.3	0.85 (0.80–0.91)	0.87 (0.81–0.92)		1.10 (1.03–1.18)	1.11 (1.04–1.19)
	Heavy	39,096	574	2.3	1.08 (0.99–1.17)	1.10 (1.01–1.20)		1.37 (1.25–1.49)	1.38 (1.26–1.51)
Moderate	Non	36,329	832	3.7	1.24 (1.15–1.33)	1.21 (1.12–1.30)		1.50 (1.37–1.65)	1.45 (1.31–1.61)
	Mild	151,249	1,474	1.5	0.92 (0.88–0.98)	0.93 (0.88–0.99)		1.15 (1.06–1.25)	1.15 (1.06–1.25)
	Moderate	168,501	1,072	1.0	0.79 (0.74–0.84)	0.79 (0.74–0.84)		1 (Ref.)	1 (Ref.)
	Heavy	75,124	724	1.5	0.95 (0.88–1.03)	0.96 (0.89–1.04)		1.18 (1.07–1.30)	1.18 (1.07–1.30)
Heavy	Non	22,604	683	4.9	1.41 (1.31–1.52)	1.38 (1.27–1.50)		1.30 (1.18–1.42)	1.26 (1.14–1.39)
	Mild	49,657	783	2.5	1.15 (1.07–1.23)	1.15 (1.06–1.24)		1.06 (0.98–1.16)	1.05 (0.96–1.15)
	Moderate	83,299	831	1.6	0.94 (0.88–1.01)	0.93 (0.87–1.00)		0.88 (0.81–0.95)	0.86 (0.79–0.94)

© 2023 Jeon KH et al. *JAMA Network Open.*

Alcohol consumption status		Subjects (N)	Events (N)	IR (per 1000 PYs)	**Non-drinkers as a reference**			**Sustained drinking at the same level as a reference**	
2009	2011				aHR[a] (95% CI)	aSHR[a] (95% CI)		aHR[a] (95% CI)	aSHR[a] (95% CI)
	Heavy	140,915	1,797	2.0	1.08 (1.03–1.13)	1.09 (1.04–1.15)		1 (Ref.)	1 (Ref.)
Vascular dementia									
Non-drinker	Non	1,857,920	6,791	0.6	1 (Ref.)	1 (Ref.)		1 (Ref.)	1 (Ref.)
	Mild	243,958	507	0.3	0.95 (0.86–1.04)	0.94 (0.85–1.03)		0.95 (0.87–1.05)	0.95 (0.86–1.04)
	Moderate	34,821	90	0.4	1.07 (0.87–1.32)	1.08 (0.88–1.34)		1.09 (0.88–1.34)	1.10 (0.89–1.37)
	Heavy	20,427	69	0.5	1.21 (0.96–1.54)	1.26 (0.99–1.60)		1.23 (0.97–1.57)	1.28 (1.01–1.63)
Mild	Non	253,643	723	0.5	1.09 (1.01–1.18)	1.09 (1.01–1.18)		1.33 (1.20–1.48)	1.33 (1.20–1.48)
	Mild	625,723	922	0.2	0.84 (0.78–0.90)	0.84 (0.78–0.91)		1 (Ref.)	1 (Ref.)
	Moderate	130,116	219	0.3	0.90 (0.79–1.04)	0.89 (0.78–1.03)		1.06 (0.92–1.23)	1.04 (0.89–1.21)
	Heavy	39,096	120	0.5	1.27 (1.05–1.52)	1.25 (1.04–1.52)		1.48 (1.22–1.79)	1.45 (1.19–1.77)
Moderate	Non	36,329	140	0.6	1.35 (1.14–1.60)	1.34 (1.13–1.59)		1.27 (1.03–1.57)	1.23 (0.99–1.52)
	Mild	151,249	260	0.3	0.89 (0.78–1.01)	0.91 (0.80–1.03)		0.84 (0.71–0.99)	0.84 (0.71–0.99)
	Moderate	168,501	295	0.3	1.06 (0.94–1.20)	1.08 (0.96–1.23)		1 (Ref.)	1 (Ref.)
	Heavy	75,124	159	0.3	1.08 (0.92–1.27)	1.08 (0.92–1.28)		1.03 (0.85–1.25)	1.01 (0.83–1.23)
Heavy	Non	22,604	109	0.8	1.46 (1.21–1.77)	1.39 (1.14–1.70)		1.39 (1.11–1.74)	1.33 (1.05–1.68)
	Mild	49,657	135	0.4	1.12 (0.94–1.33)	1.17 (0.98–1.39)		1.05 (0.86–1.28)	1.10 (0.90–1.35)
	Moderate	83,299	202	0.4	1.19 (1.03–1.38)	1.20 (1.04–1.39)		1.10 (0.92–1.31)	1.11 (0.93–1.33)
	Heavy	140,915	344	0.4	1.09 (0.97–1.22)	1.08 (0.96–1.22)		1 (Ref.)	1 (Ref.)

Abbreviation: IR, Incidence rate; HR, hazard ratio; SHR, subdistribution of hazard ratio; CI, confidence interval.

[a] adjusted for age, sex, smoking status, physical activity, area of residence, income, BMI, hypertension, diabetes mellitus, dyslipidemia, systolic blood pressure, fasting glucose level, total cholesterol level, and serum creatinine.

© 2023 Jeon KH et al. *JAMA Network Open.*

eTable 6. Hazard Ratios (HR) and 95% Confidence Intervals (CI) for the Association Between Change in Alcohol Consumption Amount and the Risk of Dementia Among Males

Alcohol consumption status		Subjects (N)	Events (N)	IR (per 1000 PYs)	Non-drinkers as a reference				Sustained drinking at the same level as a reference		
2009	2011				Crude Model	Model 1[a] HR (95% CI)	Model 2[b] HR (95% CI)		Crude Model	Model 1[a] HR (95% CI)	Model 2[b] HR (95% CI)
**All-cause dementia**											
Non-drinker	Non	477,073	15,471	5.2	1 (Ref.)	1 (Ref.)	1 (Ref.)		1 (Ref.)	1 (Ref.)	1 (Ref.)
	Mild	118,899	2,289	3.1	0.59 (0.57–0.62)	0.96 (0.92–1.00)	0.95 (0.91–0.99)		0.59 (0.57–0.62)	0.95 (0.91–0.99)	0.95 (0.90–0.99)
	Moderate	27,883	583	3.3	0.65 (0.60–0.71)	1.16 (1.07–1.26)	1.15 (1.05–1.25)		0.65 (0.60–0.71)	1.15 (1.06–1.25)	1.14 (1.05–1.24)
	Heavy	18,302	509	4.5	0.85 (0.78–0.93)	1.34 (1.22–1.47)	1.30 (1.19–1.42)		0.85 (0.78–0.93)	1.33 (1.22–1.46)	1.30 (1.19–1.42)
Mild	Non	123,471	3,181	4.1	0.80 (0.77–0.83)	1.04 (0.99–1.08)	1.03 (0.99–1.07)		2.21 (2.11–2.31)	1.30 (1.24–1.36)	1.30 (1.24–1.36)
	Mild	444,476	5,255	1.9	0.36 (0.35–0.37)	0.79 (0.77–0.82)	0.79 (0.76–0.82)		1 (Ref.)	1 (Ref.)	1 (Ref.)
	Moderate	115,114	1,327	1.8	0.35 (0.33–0.37)	0.86 (0.81–0.91)	0.85 (0.81–0.91)		0.97 (0.92–1.04)	1.09 (1.03–1.16)	1.08 (1.02–1.15)
	Heavy	35,881	725	3.2	0.63 (0.58–0.67)	1.14 (1.06–1.23)	1.12 (1.04–1.21)		1.72 (1.59–1.86)	1.43 (1.32–1.55)	1.40 (1.30–1.52)
Moderate	Non	28,663	881	4.9	0.94 (0.87–1.00)	1.26 (1.18–1.35)	1.25 (1.17–1.34)		3.37 (3.09–3.67)	1.47 (1.34–1.60)	1.47 (1.34–1.60)
	Mild	133,392	1,763	2.1	0.41 (0.39–0.43)	0.93 (0.89–0.98)	0.93 (0.88–0.98)		1.48 (1.38–1.58)	1.10 (1.03–1.18)	1.10 (1.03–1.19)
	Moderate	159,435	1,443	1.4	0.28 (0.26–0.29)	0.84 (0.79–0.89)	0.83 (0.79–0.88)		1 (Ref.)	1 (Ref.)	1 (Ref.)
	Heavy	72,255	950	2.1	0.40 (0.38–0.43)	0.99 (0.93–1.06)	0.97 (0.91–1.04)		1.45 (1.34–1.58)	1.17 (1.07–1.27)	1.16 (1.06–1.26)
Heavy	Non	19,978	809	6.6	1.27 (1.18–1.36)	1.49 (1.38–1.60)	1.45 (1.35–1.56)		2.44 (2.25–2.65)	1.36 (1.25–1.48)	1.35 (1.24–1.46)
	Mild	45,378	943	3.3	0.64 (0.60–0.68)	1.13 (1.06–1.21)	1.11 (1.04–1.19)		1.23 (1.14–1.33)	1.03 (0.95–1.11)	1.03 (0.95–1.12)
	Moderate	79,968	1,122	2.2	0.43 (0.41–0.46)	1.01 (0.95–1.07)	0.99 (0.93–1.05)		0.84 (0.78–0.90)	0.91 (0.85–0.98)	0.92 (0.86–0.99)
	Heavy	137,780	2,330	2.7	0.52 (0.50–0.54)	1.10 (1.05–1.15)	1.08 (1.03–1.13)		1 (Ref.)	1 (Ref.)	1 (Ref.)
**Alzheimer's disease**											
Non-drinker	Non	477,073	12,174	4.1	1 (Ref.)	1 (Ref.)	1 (Ref.)		1 (Ref.)	1 (Ref.)	1 (Ref.)
	Mild	118,899	1,760	2.4	0.58 (0.55–0.61)	0.96 (0.91–1.01)	0.96 (0.91–1.01)		0.58 (0.55–0.61)	0.94 (0.90–0.99)	0.94 (0.90–0.99)
	Moderate	27,883	442	2.5	0.63 (0.57–0.69)	1.15 (1.05–1.27)	1.15 (1.04–1.26)		0.63 (0.57–0.69)	1.14 (1.03–1.25)	1.13 (1.03–1.25)
	Heavy	18,302	393	3.4	0.83 (0.75–0.92)	1.34 (1.21–1.48)	1.31 (1.18–1.45)		0.83 (0.75–0.92)	1.33 (1.20–1.47)	1.30 (1.18–1.45)
Mild	Non	123,471	2,408	3.1	0.77 (0.74–0.80)	1.00 (0.96–1.05)	1.00 (0.96–1.05)		2.22 (2.11–2.34)	1.27 (1.21–1.34)	1.27 (1.20–1.34)
	Mild	444,476	3,944	1.4	0.35 (0.33–0.36)	0.78 (0.75–0.81)	0.78 (0.75–0.81)		1 (Ref.)	1 (Ref.)	1 (Ref.)
	Moderate	115,114	994	1.4	0.34 (0.32–0.36)	0.86 (0.81–0.92)	0.86 (0.81–0.92)		0.98 (0.91–1.05)	1.11 (1.03–1.19)	1.11 (1.03–1.19)
	Heavy	35,881	537	2.4	0.59 (0.54–0.64)	1.11 (1.01–1.21)	1.10 (1.01–1.20)		1.70 (1.55–1.87)	1.41 (1.29–1.55)	1.39 (1.27–1.53)
Moderate	Non	28,663	691	3.9	0.93 (0.86–1.00)	1.27 (1.17–1.37)	1.26 (1.17–1.37)		3.74 (3.38–4.13)	1.54 (1.39–1.70)	1.52 (1.38–1.69)
	Mild	133,392	1,345	1.6	0.40 (0.38–0.42)	0.94 (0.89–0.99)	0.94 (0.89–0.99)		1.60 (1.47–1.74)	1.16 (1.07–1.27)	1.16 (1.07–1.26)
	Moderate	159,435	1,017	1.0	0.25 (0.23–0.27)	0.79 (0.74–0.85)	0.80 (0.74–0.85)		1 (Ref.)	1 (Ref.)	1 (Ref.)
	Heavy	72,255	695	1.5	0.37 (0.35–0.40)	0.96 (0.89–1.04)	0.95 (0.88–1.03)		1.51 (1.37–1.66)	1.18 (1.07–1.31)	1.17 (1.06–1.30)
Heavy	Non	19,978	624	5.1	1.25 (1.15–1.36)	1.48 (1.37–1.61)	1.45 (1.34–1.57)		2.50 (2.27–2.74)	1.36 (1.23–1.49)	1.33 (1.21–1.47)
	Mild	45,378	729	2.6	0.62 (0.58–0.67)	1.14 (1.05–1.23)	1.12 (1.04–1.21)		1.25 (1.14–1.37)	1.03 (0.95–1.13)	1.03 (0.94–1.13)
	Moderate	79,968	808	1.6	0.39 (0.37–0.42)	0.96 (0.89–1.03)	0.95 (0.89–1.03)		0.79 (0.73–0.86)	0.87 (0.80–0.95)	0.87 (0.80–0.95)

© 2023 Jeon KH et al. *JAMA Network Open.*

Alcohol consumption status		Subjects (N)	Events (N)	IR (per 1000 PYs)	**Non-drinkers as a reference**			**Sustained drinking at the same level as a reference**		
2009	2011				Crude Model	Model 1[a] HR (95% CI)	Model 2[b] HR (95% CI)	Crude Model	Model 1[a] HR (95% CI)	Model 2[b] HR (95% CI)
	Heavy	137,780	1,763	2.0	0.50 (0.48–0.53)	1.10 (1.05–1.16)	1.09 (1.04–1.15)	1 (Ref.)	1 (Ref.)	1 (Ref.)
**Vascular dementia**										
Non-drinker	Non	477,073	1,747	0.6	1 (Ref.)	1 (Ref.)	1 (Ref.)	1 (Ref.)	1 (Ref.)	1 (Ref.)
	Mild	118,899	297	0.4	0.68 (0.60–0.77)	0.99 (0.88–1.13)	0.98 (0.86–1.11)	0.68 (0.60–0.77)	0.99 (0.88–1.13)	0.98 (0.86–1.11)
	Moderate	27,883	80	0.5	0.80 (0.64–1.01)	1.24 (0.99–1.55)	1.16 (0.93–1.46)	0.80 (0.64–1.01)	1.24 (0.99–1.56)	1.17 (0.93–1.47)
	Heavy	18,302	61	0.5	0.95 (0.73–1.22)	1.32 (1.02–1.70)	1.21 (0.93–1.56)	0.95 (0.73–1.22)	1.32 (1.02–1.70)	1.22 (0.94–1.58)
Mild	Non	123,471	414	0.5	0.94 (0.84–1.04)	1.16 (1.04–1.30)	1.15 (1.03–1.28)	2.04 (1.80–2.30)	1.37 (1.21–1.55)	1.38 (1.22–1.56)
	Mild	444,476	747	0.3	0.46 (0.42–0.50)	0.86 (0.78–0.94)	0.84 (0.77–0.92)	1 (Ref.)	1 (Ref.)	1 (Ref.)
	Moderate	115,114	199	0.3	0.46 (0.40–0.54)	0.91 (0.79–1.06)	0.87 (0.74–1.01)	1.01 (0.86–1.19)	1.07 (0.91–1.25)	1.03 (0.88–1.21)
	Heavy	35,881	116	0.5	0.87 (0.72–1.05)	1.37 (1.12–1.66)	1.27 (1.05–1.54)	1.89 (1.55–2.31)	1.58 (1.29–1.94)	1.50 (1.22–1.83)
Moderate	Non	28,663	117	0.7	1.13 (0.93–1.37)	1.42 (1.18–1.72)	1.37 (1.13–1.65)	2.32 (1.86–2.88)	1.25 (0.99–1.57)	1.30 (1.03–1.63)
	Mild	133,392	233	0.3	0.49 (0.43–0.56)	0.92 (0.80–1.06)	0.88 (0.76–1.01)	1.00 (0.84–1.19)	0.82 (0.69–0.98)	0.84 (0.70–1.00)
	Moderate	159,435	281	0.3	0.49 (0.43–0.55)	1.12 (0.98–1.28)	1.05 (0.92–1.19)	1 (Ref.)	1 (Ref.)	1 (Ref.)
	Heavy	72,255	154	0.3	0.58 (0.49–0.69)	1.15 (0.97–1.36)	1.06 (0.90–1.26)	1.19 (0.98–1.46)	1.03 (0.85–1.26)	1.02 (0.83–1.24)
Heavy	Non	19,978	103	0.8	1.42 (1.16–1.74)	1.60 (1.30–1.96)	1.51 (1.23–1.85)	2.14 (1.70–2.68)	1.37 (1.09–1.73)	1.40 (1.11–1.77)
	Mild	45,378	131	0.5	0.82 (0.69–0.98)	1.27 (1.06–1.52)	1.19 (0.99–1.42)	1.24 (1.01–1.52)	1.09 (0.89–1.33)	1.11 (0.91–1.36)
	Moderate	79,968	197	0.4	0.68 (0.59–0.79)	1.29 (1.11–1.49)	1.19 (1.02–1.39)	1.02 (0.85–1.22)	1.10 (0.92–1.31)	1.11 (0.93–1.33)
	Heavy	137,780	339	0.4	0.67 (0.59–0.75)	1.17 (1.04–1.32)	1.07 (0.95–1.21)	1 (Ref.)	1 (Ref.)	1 (Ref.)

Abbreviation: IR, Incidence rate; HR, hazard ratio; CI, confidence interval.

[a] Model 1: adjusted for age, sex, smoking status, physical activity, area of residence, and income

[b] Model 2: Model 1 plus BMI, hypertension, diabetes mellitus, dyslipidemia, systolic blood pressure, fasting glucose level, total cholesterol level, and serum creatinine.

© 2023 Jeon KH et al. *JAMA Network Open.*

eTable 7. Hazard Ratios (HR) and 95% Confidence Intervals (CI) for the Association Between Change in Alcohol Consumption Amount and the Risk of Dementia Among Females

Alcohol consumption status		Subjects (N)	Events (N)	IR (per 1000 PYs)	**Non-drinkers as a reference**			**Sustained drinking at the same level as a reference**		
2009	2011				Crude Model	Model 1[a] HR (95% CI)	Model 2[b] HR (95% CI)	Crude Model	Model 1[a] HR (95% CI)	Model 2[b] HR (95% CI)
**All-cause dementia**										
Non-drinker	Non	1,380,847	53,208	6.1	1 (Ref.)	1 (Ref.)	1 (Ref.)	1 (Ref.)	1 (Ref.)	1 (Ref.)
	Mild	125,059	2,049	2.6	0.43 (0.41–0.45)	0.90 (0.86–0.94)	0.91 (0.87–0.95)	0.43 (0.41–0.45)	0.89 (0.85–0.93)	0.90 (0.86–0.95)
	Moderate	6,938	135	3.1	0.49 (0.41–0.58)	1.04 (0.88–1.24)	1.04 (0.88–1.24)	0.49 (0.41–0.58)	1.04 (0.87–1.23)	1.04 (0.87–1.24)
	Heavy	2,125	47	3.5	0.58 (0.4–0.77)	1.34 (1.00–1.79)	1.37 (1.02–1.82)	0.58 (0.43–0.77)	1.33 (0.99–1.77)	1.36 (1.02–1.81)
Mild	Non	130,172	2,972	3.6	0.59 (0.56–0.61)	1.01 (0.97–1.05)	1.02 (0.98–1.06)	2.86 (2.68–3.05)	1.26 (1.18–1.34)	1.25 (1.17–1.34)
	Mild	181,247	1,435	1.2	0.21 (0.19–0.22)	0.77 (0.73–0.81)	0.78 (0.74–0.83)	1 (Ref.)	1 (Ref.)	1 (Ref.)
	Moderate	15,002	144	1.5	0.25 (0.21–0.29)	0.90 (0.76–1.06)	0.91 (0.77–1.08)	1.20 (1.01–1.43)	1.17 (0.98–1.39)	1.16 (0.97–1.38)
	Heavy	3,215	42	2.1	0.35 (0.26–0.47)	0.94 (0.69–1.27)	0.94 (0.69–1.27)	1.69 (1.24–2.30)	1.19 (0.87–1.61)	1.17 (0.86–1.59)
Moderate	Non	7,666	179	3.7	0.61 (0.53–0.71)	1.15 (0.99–1.34)	1.16 (0.99–1.34)	2.71 (2.07–3.54)	1.21 (0.92–1.60)	1.25 (0.95–1.65)
	Mild	17,857	166	1.5	0.24 (0.20–0.28)	0.88 (0.75–1.03)	0.89 (0.76–1.04)	1.05 (0.80–1.38)	0.99 (0.76–1.30)	1.02 (0.78–1.35)
	Moderate	9,066	78	1.4	0.23 (0.18–0.28)	0.88 (0.70–1.10)	0.87 (0.70–1.09)	1 (Ref.)	1 (Ref.)	1 (Ref.)
	Heavy	2,869	36	2.0	0.32 (0.23–0.44)	1.19 (0.85–1.67)	1.19 (0.85–1.67)	1.39 (0.93–2.09)	1.38 (0.92–2.07)	1.40 (0.94–2.10)
Heavy	Non	2,626	74	4.5	0.71 (0.56–0.90)	1.25 (0.99–1.58)	1.27 (1.00–1.60)	1.96 (1.34–2.87)	0.96 (0.65–1.43)	0.96 (0.65–1.43)
	Mild	4,279	62	2.3	0.38 (0.30–0.49)	1.29 (1.00–1.65)	1.30 (1.01–1.67)	1.06 (0.71–1.56)	1.07 (0.72–1.59)	1.07 (0.72–1.58)
	Moderate	3,331	31	1.5	0.25 (0.17–0.35)	0.99 (0.69–1.40)	0.99 (0.70–1.41)	0.69 (0.43–1.09)	0.84 (0.53–1.33)	0.84 (0.53–1.34)
	Heavy	3,135	43	2.2	0.36 (0.27–0.49)	1.19 (0.88–1.60)	1.19 (0.88–1.60)	1 (Ref.)	1 (Ref.)	1 (Ref.)
**Alzheimer's disease**										
Non-drinker	Non	1,380,847	43,652	5.0	1 (Ref.)	1 (Ref.)	1 (Ref.)	1 (Ref.)	1 (Ref.)	1 (Ref.)
	Mild	125,059	1,657	2.1	0.42 (0.40–0.44)	0.90 (0.85–0.94)	0.91 (0.86–0.96)	0.42 (0.40–0.44)	0.89 (0.85–0.94)	0.90 (0.86–0.95)
	Moderate	6,938	117	2.7	0.52 (0.43–0.63)	1.13 (0.94–1.36)	1.13 (0.94–1.37)	0.52 (0.43–0.63)	1.12 (0.93–1.35)	1.13 (0.94–1.36)
	Heavy	2,125	32	2.4	0.48 (0.33–0.68)	1.13 (0.79–1.61)	1.15 (0.81–1.64)	0.48 (0.33–0.68)	1.12 (0.79–1.59)	1.14 (0.80–1.63)
Mild	Non	130,172	2,411	2.9	0.58 (0.56–0.60)	1.01 (0.97–1.05)	1.02 (0.97–1.06)	2.96 (2.76–3.19)	1.26 (1.17–1.36)	1.26 (1.17–1.35)
	Mild	181,247	1,122	1.0	0.20 (0.18–0.21)	0.76 (0.71–0.80)	0.77 (0.72–0.82)	1 (Ref.)	1 (Ref.)	1 (Ref.)
	Moderate	15,002	110	1.2	0.23 (0.19–0.28)	0.87 (0.72–1.06)	0.89 (0.73–1.07)	1.18 (0.97–1.44)	1.16 (0.95–1.42)	1.16 (0.95–1.42)
	Heavy	3,215	37	1.8	0.37 (0.27–0.51)	1.03 (0.75–1.42)	1.03 (0.75–1.43)	1.91 (1.37–2.64)	1.33 (0.96–1.85)	1.32 (0.95–1.83)
Moderate	Non	7,666	141	2.9	0.59 (0.50–0.70)	1.13 (0.96–1.34)	1.14 (0.97–1.35)	3.07 (2.24–4.20)	1.33 (0.97–1.83)	1.39 (1.01–1.91)
	Mild	17,857	129	1.1	0.23 (0.19–0.27)	0.87 (0.73–1.04)	0.88 (0.74–1.05)	1.17 (0.85–1.61)	1.12 (0.81–1.54)	1.16 (0.84–1.60)
	Moderate	9,066	54	0.9	0.19 (0.15–0.25)	0.78 (0.60–1.02)	0.77 (0.59–1.01)	1 (Ref.)	1 (Ref.)	1 (Ref.)
	Heavy	2,869	28	1.5	0.31 (0.21–0.45)	1.20 (0.82–1.75)	1.21 (0.83–1.76)	1.58 (0.99–2.50)	1.58 (0.99–2.52)	1.61 (1.01–2.56)
Heavy	Non	2,626	60	3.6	0.72 (0.55–0.93)	1.27 (0.98–1.64)	1.29 (0.99–1.67)	2.06 (1.35–3.14)	0.97 (0.62–1.50)	0.95 (0.61–1.48)
	Mild	4,279	54	2.0	0.40 (0.31–0.53)	1.42 (1.08–1.85)	1.43 (1.09–1.87)	1.16 (0.76–1.79)	1.19 (0.77–1.84)	1.15 (0.75–1.79)
	Moderate	3,331	23	1.1	0.22 (0.15–0.34)	0.94 (0.62–1.41)	0.95 (0.63–1.42)	0.64 (0.38–1.09)	0.81 (0.47–1.37)	0.79 (0.46–1.35)

© 2023 Jeon KH et al. *JAMA Network Open.*

Alcohol consumption status		Subjects (N)	Events (N)	IR (per 1000 PYs)	**Non-drinkers as a reference**			**Sustained drinking at the same level as a reference**		
2009	2011				Crude Model	Model 1[a] HR (95% CI)	Model 2[b] HR (95% CI)	Crude Model	Model 1[a] HR (95% CI)	Model 2[b] HR (95% CI)
	Heavy	3,135	34	1.7	0.35 (0.25–0.49)	1.19 (0.85–1.66)	1.19 (0.85–1.66)	1 (Ref.)	1 (Ref.)	1 (Ref.)
**Vascular dementia**										
Non-drinker	Non	1,380,847	5,044	0.6	1 (Ref.)	1 (Ref.)	1 (Ref.)	1 (Ref.)	1 (Ref.)	1 (Ref.)
	Mild	125,059	211	0.3	0.45 (0.39–0.52)	0.88 (0.76–1.01)	0.89 (0.78–1.03)	0.45 (0.39–0.52)	0.88 (0.76–1.01)	0.89 (0.77–1.03)
	Moderate	6,938	10	0.2	0.37 (0.19–0.70)	0.69(0.36–1.33)	0.68 (0.36–1.32)	0.37 (0.19–0.70)	0.70 (0.36–1.34)	0.69 (0.36–1.32)
	Heavy	2,125	8	0.6	1.06 (0.53–2.12)	2.10 (1.05–4.20)	2.10 (1.05–4.20)	1.06 (0.53–2.12)	2.11 (1.05–4.22)	2.11 (1.05–4.22)
Mild	Non	130,172	309	0.4	0.64 (0.57–0.72)	1.04 (0.93–1.18)	1.06 (0.94–1.19)	2.45 (2.03–2.7)	1.29 (1.06–1.57)	1.29 (1.06–1.57)
	Mild	181,247	174	0.2	0.26 (0.22–0.30)	0.82 (0.70–0.95)	0.83 (0.71–0.97)	1 (Ref.)	1 (Ref.)	1 (Ref.)
	Moderate	15,002	20	0.2	0.36 (0.23–0.56)	1.04 (0.66–1.64)	1.04 (0.66–1.63)	1.36 (0.85–2.19)	1.25 (0.77–2.01)	1.21 (0.75–1.95)
	Heavy	3,215	4	0.2	0.35 (0.13–0.93)	0.81 (0.30–2.15)	0.79 (0.30–2.11)	1.33 (0.49–3.59)	0.95 (0.35–2.57)	0.91 (0.34–2.47)
Moderate	Non	7,666	23	0.5	0.81 (0.53–1.23)	1.40 (0.92–2.13)	1.40 (0.92–2.12)	1.88 (0.96–3.67)	0.98 (0.49–1.95)	1.00 (0.50–2.00)
	Mild	17,857	27	0.2	0.41 (0.28–0.60)	1.21 (0.82–1.78)	1.21 (0.82–1.78)	0.94 (0.49–1.81)	0.87 (0.45–1.68)	0.91 (0.47–1.76)
	Moderate	9,066	15	0.3	0.43 (0.26–0.73)	1.33 (0.79–2.26)	1.29 (0.76–2.19)	1 (Ref.)	1 (Ref.)	1 (Ref.)
	Heavy	2,869	5	0.3	0.39 (0.15–1.04)	1.14 (0.43–3.05)	1.11 (0.42–2.95)	0.90 (0.30–2.74)	0.87 (0.29–2.64)	0.89 (0.29–2.71)
Heavy	Non	2,626	6	0.4	0.54 (0.22–1.29)	0.87 (0.36–2.10)	0.88 (0.36–2.11)	1.21 (0.35–4.19)	0.78 (0.21–2.85)	0.80 (0.22–2.95)
	Mild	4,279	4	0.1	0.26 (0.10–0.70)	0.71 (0.27–1.89)	0.70 (0.26–1.87)	0.59 (0.16–2.21)	0.60 (0.16–2.27)	0.63 (0.17–2.40)
	Moderate	3,331	5	0.2	0.42 (0.18–1.01)	1.26 (0.52–3.02)	1.21 (0.50–2.92)	0.95 (0.28–3.28)	1.02 (0.30–3.53)	1.06 (0.30–3.71)
	Heavy	3,135	5	0.3	0.44 (0.18–1.06)	1.16 (0.48–2.78)	1.12 (0.47–2.70)	1 (Ref.)	1 (Ref.)	1 (Ref.)

Abbreviation: IR, Incidence rate; HR, hazard ratio; CI, confidence interval.

[a] Model 1: adjusted for age, sex, smoking status, physical activity, area of residence, and income

[b] Model 2: Model 1 plus BMI, hypertension, diabetes mellitus, dyslipidemia, systolic blood pressure, fasting glucose level, total cholesterol level, and serum creatinine.

© 2023 Jeon KH et al. *JAMA Network Open.*

eTable 8. Hazard Ratios and 95% Confidence Intervals for Dementia According to Change in Drinking Level by Age

		Non-drinkers as a reference					Sustained drinking at the same level as a reference			
2009 \ 2011		Non	Mild	Moderate	Heavy		Non	Mild	Moderate	Heavy
**All-cause dementia**										
<65 years	Non	1 (Ref.)	0.90 (0.84–0.96)	1.16 (1.01–1.33)	1.36 (1.15–1.60)		1 (Ref.)	0.91 (0.85–0.97)	1.18 (1.02–1.36)	1.38 (1.16–1.63)
	Mild	1.02 (0.96–1.08)	0.78 (0.74–0.82)	0.88(0.80–0.97)	1.30 (1.14–1.48)		1.31 (1.22–1.42)	1 (Ref.)	1.13 (1.02–1.25)	1.67 (1.46–1.91)
	Moderate	1.33 (1.17–1.50)	0.93 (0.85–1.01)	0.86 (0.78–0.93)	1.01 (0.90–1.13)		1.63 (1.39–1.90)	1.10 (0.98–1.23)	1 (Ref.)	1.19 (1.04–1.37)
	Heavy	1.62 (1.41–1.87)	1.14 (1.01–1.29)	1.01 (0.90–1.12)	1.13 (1.05–1.23)		1.47 (1.24–1.73)	1.01 (0.88–1.17)	0.89 (0.79–1.01)	1 (Ref.)
≥65 years	Non	1 (Ref.)	0.97 (0.94–1.00)	1.11 (1.02–1.22)	1.28 (1.16–1.41)		1 (Ref.)	0.96 (0.93–1.00)	1.11 (1.01–1.21)	1.28 (1.16–1.41)
	Mild	1.05 (1.02–1.08)	0.83 (0.81–0.86)	0.88 (0.82–0.94)	1.04 (0.96–1.14)		1.24 (1.18–1.29)	1 (Ref.)	1.06 (0.99–1.14)	1.24 (1.13–1.36)
	Moderate	1.22 (1.13–1.30)	0.94 (0.89–1.00)	0.86 (0.80–0.92)	0.99 (0.92–1.07)		1.38 (1.25–1.52)	1.09 (1.00–1.19)	1 (Ref.)	1.14 (1.03–1.26)
	Heavy	1.37 (1.27–1.48)	1.14 (1.06–1.23)	1.00 (0.93–1.08)	1.06 (1.01–1.12)		1.28 (1.17–1.40)	1.07 (0.98–1.17)	0.94 (0.86–1.02)	1 (Ref.)
**Alzheimer's disease**										
<65 years	Non	1 (Ref.)	0.90 (0.83–0.97)	1.16 (0.98–1.38)	1.39 (1.14–1.71)		1 (Ref.)	0.90 (0.83–0.98)	1.17 (0.98–1.39)	1.40 (1.14–1.72)
	Mild	1.01 (0.94–1.08)	0.76 (0.72–0.81)	0.83 (0.74–0.94)	1.30 (1.11–1.53)		1.33 (1.21–1.45)	1 (Ref.)	1.10 (0.97–1.25)	1.73 (1.46–2.04)
	Moderate	1.35 (1.16–1.57)	0.89 (0.80–0.99)	0.77 (0.69–0.86)	0.99 (0.86–1.14)		1.85 (1.53–2.24)	1.17 (1.01–1.35)	1 (Ref.)	1.30 (1.09–1.54)
	Heavy	1.64 (1.38–1.95)	1.19 (1.02–1.38)	0.97 (0.85–1.11)	1.15 (1.05–1.27)		1.46 (1.20–1.78)	1.04 (0.87–1.23)	0.84 (0.72–0.98)	1 (Ref.)
≥65 years	Non	1 (Ref.)	0.97 (0.93–1.01)	1.14 (1.03–1.25)	1.28 (1.15–1.43)		1 (Ref.)	0.96 (0.92–1.00)	1.13 (1.02–1.24)	1.27 (1.14–1.42)
	Mild	1.03 (1.00–1.06)	0.83 (0.80–0.86)	0.90 (0.84–0.97)	1.03 (0.93–1.14)		1.21 (1.15–1.27)	1 (Ref.)	1.10 (1.02–1.18)	1.24 (1.12–1.37)
	Moderate	1.23 (1.13–1.32)	0.98 (0.92–1.04)	0.85 (0.79–0.92)	0.98 (0.90–1.07)		1.41 (1.26–1.57)	1.14 (1.04–1.26)	1 (Ref.)	1.13 (1.01–1.27)
	Heavy	1.36 (1.25–1.48)	1.16 (1.07–1.26)	0.97 (0.89–1.05)	1.07 (1.02–1.14)		1.26 (1.13–1.39)	1.08 (0.98–1.19)	0.90 (0.82–0.99)	1 (Ref.)
**Vascular dementia**										
<65 years	Non	1 (Ref.)	0.92 (0.78–1.07)	1.29 (0.95–1.74)	1.23 (0.83–1.80)		1 (Ref.)	0.93 (0.79–1.08)	1.29 (0.95–1.75)	1.23 (0.83–1.81)
	Mild	1.08 (0.93–1.24)	0.84 (0.75–0.94)	0.97 (0.80–1.18)	1.20 (0.90–1.62)		1.29 (1.09–1.53)	1 (Ref.)	1.15 (0.93–1.41)	1.42 (1.05–1.91)
	Moderate	1.34 (1.00–1.79)	1.04 (0.87–1.24)	1.09 (0.92–1.29)	1.05 (0.83–1.33)		1.17 (0.83–1.64)	0.95 (0.76–1.18)	1 (Ref.)	0.97 (0.75–1.27)
	Heavy	1.58 (1.13–2.20)	1.12 (0.85–1.47)	1.13 (0.91–1.41)	1.07 (0.90–1.28)		1.53 (1.05–2.24)	1.05 (0.77–1.43)	1.06 (0.82–1.36)	1 (Ref.)
≥65 years	Non	1 (Ref.)	0.99 (0.88–1.10)	0.92 (0.68–1.23)	1.21 (0.89–1.64)		1 (Ref.)	0.99 (0.88–1.11)	0.92 (0.68–1.24)	1.21 (0.89–1.65)
	Mild	1.12 (1.02–1.23)	0.86 (0.78–0.95)	0.83 (0.68–1.02)	1.31 (1.04–1.66)		1.33 (1.17–1.52)	1 (Ref.)	0.96 (0.78–1.20)	1.51 (1.18–1.94)
	Moderate	1.37 (1.11–1.69)	0.74 (0.61–0.90)	1.00 (0.83–1.21)	1.10 (0.88–1.38)		1.29 (0.97–1.71)	0.73 (0.57–0.95)	1 (Ref.)	1.09 (0.82–1.45)
	Heavy	1.41 (1.12–1.78)	1.13 (0.90–1.41)	1.23 (1.01–1.50)	1.09 (0.93–1.27)		1.32 (1.00–1.75)	1.05 (0.81–1.37)	1.15 (0.90–1.46)	1(Ref.)

Adjusted for age, sex, smoking status, physical activity, area of residence, income, BMI, hypertension, diabetes mellitus, dyslipidemia, systolic blood pressure, fasting glucose level, total cholesterol level, and serum creatinine.

© 2023 Jeon KH et al. *JAMA Network Open.*

eTable 9. Hazard Ratios and 95% Confidence Intervals for Dementia According to Change in Drinking Level by Smoking

		**Non-drinkers as a reference**				**Sustained drinking at the same level as a reference**			
2009 \ 2011		Non	Mild	Moderate	Heavy	Non	Mild	Moderate	Heavy
**All-cause dementia**									
Non-smoker	Non	1 (Ref.)	1.03 (0.95–1.11)	1.20 (1.05–1.37)	1.25 (1.08–1.45)	1 (Ref.)	1.02 (0.94–1.11)	1.19 (1.04–1.36)	1.26 (1.08–1.46)
	Mild	1.05 (0.97–1.13)	0.83 (0.78–0.88)	0.93 (0.85–1.02)	1.23 (1.09–1.38)	1.23 (1.13–1.35)	1 (Ref.)	1.13 (1.02–1.25)	1.47 (1.30–1.67)
	Moderate	1.27 (1.10–1.47)	0.95 (0.87–1.04)	0.93 (0.85–1.01)	1.04 (0.94–1.16)	1.35 (1.15–1.59)	1.02 (0.91–1.14)	1 (Ref.)	1.11 (0.98–1.25)
	Heavy	1.57 (1.36–1.81)	1.16 (1.04–1.29)	1.03 (0.94–1.14)	1.13 (1.05–1.21)	1.42 (1.22–1.66)	1.04 (0.92–1.17)	0.92 (0.82–1.02)	1 (Ref.)
Smoker	Non	1 (Ref.)	0.92 (0.88–0.95)	1.08 (0.99–1.19)	1.33 (1.20–1.47)	1 (Ref.)	0.91 (0.88–0.94)	1.08 (0.98–1.18)	1.32 (1.19–1.46)
	Mild	1.02 (0.99–1.05)	0.78 (0.76–0.81)	0.83 (0.78–0.89)	1.03 (0.94–1.13)	1.28 (1.23–1.34)	1 (Ref.)	1.07 (0.99–1.14)	1.30 (1.18–1.43)
	Moderate	1.22 (1.14–1.31)	0.91 (0.86–0.96)	0.78 (0.73–0.84)	0.94 (0.87–1.03)	1.50 (1.36–1.65)	1.14 (1.05–1.24)	1 (Ref.)	1.19 (1.07–1.33)
	Heavy	1.38 (1.28–1.49)	1.11 (1.03–1.21)	0.96 (0.89–1.04)	1.05 (0.99–1.11)	1.30 (1.18–1.42)	1.06 (0.96–1.16)	0.92 (0.84–1.01)	1 (Ref.)
**Alzheimer's disease**									
Non-smoker	Non	1 (Ref.)	1.02 (0.93–1.12)	1.23 (1.05–1.44)	1.23 (1.04–1.47)	1 (Ref.)	1.01 (0.91–1.10)	1.21 (1.03–1.41)	1.22 (1.03–1.46)
	Mild	1.00 (0.91–1.10)	0.81 (0.76–0.87)	0.94 (0.84–1.04)	1.26 (1.10–1.44)	1.20 (1.08–1.33)	1 (Ref.)	1.16 (1.04–1.31)	1.54 (1.34–1.78)
	Moderate	1.38 (1.18–1.62)	1.00 (0.91–1.11)	0.91 (0.82–1.01)	1.05 (0.93–1.18)	1.45 (1.21–1.75)	1.08 (0.95–1.23)	1 (Ref.)	1.12 (0.97–1.30)
	Heavy	1.63 (1.38–1.93)	1.18 (1.04–1.34)	0.99 (0.88–1.11)	1.18 (1.09–1.28)	1.42 (1.19–1.69)	1.01 (0.88–1.16)	0.84 (0.74–0.95)	1 (Ref.)
Smoker	Non	1 (Ref.)	0.91 (0.88–0.95)	1.10 (0.99–1.21)	1.35 (1.20–1.51)	1 (Ref.)	0.91 (0.87–0.94)	1.08 (0.98–1.20)	1.33 (1.19–1.50)
	Mild	1.01 (0.98–1.04)	0.77 (0.75–0.80)	0.83 (0.77–0.89)	1.00 (0.90–1.11)	1.27 (1.21–1.33)	1 (Ref.)	1.08 (1.00–1.17)	1.27 (1.14–1.42)
	Moderate	1.21 (1.12–1.30)	0.90 (0.85–0.96)	0.74 (0.68–0.80)	0.92 (0.83–1.01)	1.54 (1.37–1.72)	1.19 (1.08–1.32)	1 (Ref.)	1.22 (1.08–1.38)
	Heavy	1.36 (1.25–1.48)	1.14 (1.05–1.25)	0.93 (0.85–1.02)	1.03 (0.97–1.10)	1.28 (1.15–1.42)	1.10 (0.99–1.22)	0.91 (0.82–1.01)	1 (Ref.)
**Vascular dementia**									
Non-smoker	Non	1 (Ref.)	1.10 (0.89–1.35)	1.00 (0.69–1.43)	1.27 (0.88–1.84)	1 (Ref.)	1.11 (0.90–1.36)	1.01 (0.70–1.46)	1.30 (0.89–1.88)
	Mild	1.14 (0.93–1.39)	0.90 (0.78–1.05)	0.97 (0.78–1.21)	1.15 (0.86–1.55)	1.22 (0.98–1.52)	1 (Ref.)	1.07 (0.85–1.35)	1.24 (0.91–1.69)
	Moderate	0.99 (0.65–1.50)	0.70 (0.55–0.89)	1.05 (0.87–1.27)	1.05 (0.82–1.34)	0.97 (0.62–1.53)	0.68 (0.52–0.89)	1 (Ref.)	1.00 (0.76–1.32)
	Heavy	1.56 (1.07–2.28)	1.16 (0.89–1.52)	1.25 (1.01–1.54)	1.06 (0.88–1.26)	1.50 (1.01–2.25)	1.10 (0.82–1.48)	1.17 (0.92–1.50)	1 (Ref.)
Smoker	Non	1 (Ref.)	0.91 (0.82–1.01)	1.10 (0.85–1.43)	1.17 (0.85–1.61)	1 (Ref.)	0.92 (0.83–1.02)	1.11 (0.86–1.44)	1.18 (0.86–1.62)
	Mild	1.09 (1.00–1.18)	0.81 (0.74–0.88)	0.84 (0.70–1.01)	1.34 (1.06–1.70)	1.37 (1.22–1.54)	1 (Ref.)	1.03 (0.85–1.26)	1.65 (1.29–2.11)
	Moderate	1.46 (1.22–1.76)	1.00 (0.86–1.17)	1.04 (0.88–1.23)	1.08 (0.87–1.35)	1.43 (1.11–1.84)	0.97 (0.78–1.21)	1 (Ref.)	1.06 (0.81–1.38)
	Heavy	1.44 (1.15–1.79)	1.09 (0.86–1.37)	1.12 (0.91–1.37)	1.09 (0.93–1.28)	1.32 (1.01–1.73)	1.01 (0.77–1.33)	1.03 (0.81–1.32)	1 (Ref.)

Adjusted for age, sex, smoking status, physical activity, area of residence, income, BMI, hypertension, diabetes mellitus, dyslipidemia, systolic blood pressure, fasting glucose level, total cholesterol level, and serum creatinine.

© 2023 Jeon KH et al. *JAMA Network Open.*

eTable 10. Associations Between Changes in Drinking Level From 2009 to 2013 and Dementia

Alcohol consumption status			Subjects (N)	Events (N)	IR (per 1000 PYs)	Non-drinkers as a reference		Sustained drinking at the same level as a reference
2009	2011	2013				HR (95% CI)		HR (95% CI)
**All-cause dementia**								
Non	Non	Non	1,308,226	34,615	6.1	1 (Ref.)		1(Ref.)
		Mild–Moderate	86,706	1,273	3.4	1.01 (0.95–1.07)		1.01 (0.95–1.07)
		Heavy	2,649	75	6.6	1.65 (1.31–2.07)		1.66 (1.32–2.09)
	Mild–Moderate	Non	87,657	1,462	3.8	0.99 (0.94–1.05)		0.99 (0.94–1.05)
		Mild–Moderate	114,312	1,016	2.0	0.82 (0.77–0.88)		0.82 (0.77–0.88)
		Heavy	6,416	99	3.5	1.20 (0.98–1.46)		1.20 (0.99–1.47)
	Heavy	Non	2,187	82	8.6	1.57 (1.25–1.96)		1.58 (1.26–1.97)
		Mild–Moderate	6,723	107	3.7	1.20 (0.99–1.45)		1.21 (0.99–1.46)
		Heavy	5,974	98	3.8	1.18 (0.97–1.45)		1.19 (0.97–1.46)
Mild–Moderate	Non	Non	121,702	2,596	4.9	1.10 (1.06–1.15)		1.48 (1.40–1.57)
		Mild–Moderate	89,422	1,062	2.7	0.92 (0.87–0.98)		1.24 (1.16–1.33)
		Heavy	5,032	110	5.0	1.37 (1.13–1.67)		1.81 (1.49–2.20)
	Mild–Moderate	Non	116,783	1,772	3.5	1.02 (0.97–1.08)		1.37 (1.29–1.45)
		Mild–Moderate	667,076	4,150	1.4	0.75 (0.72–0.77)		1 (Ref.)
		Heavy	49,112	434	2.0	1.01 (0.91–1.11)		1.34 (1.21–1.48)
	Heavy	Non	5,831	149	5.9	1.32 (1.12–1.55)		1.73 (1.46–2.04)
		Mild–Moderate	49,487	462	2.1	0.95 (0.86–1.04)		1.26 (1.14–1.38)
		Heavy	30,559	313	2.4	1.01 (0.90–1.13)		1.33 (1.18–1.50)
Heavy	Non	Non	4,789	192	9.3	1.51 (1.30–1.74)		1.45 (1.22–1.71)
		Mild–Moderate	6,908	161	5.4	1.27 (1.08–1.49)		1.21 (1.01–1.44)
		Heavy	4,437	104	5.4	1.36 (1.12–1.66)		1.29 (1.04–1.58)
	Mild–Moderate	Non	7,506	243	7.5	1.63 (1.43–1.85)		1.56 (1.34–1.82)
		Mild–Moderate	64,843	628	2.2	0.97 (0.89–1.05)		0.92 (0.83–1.03)
		Heavy	28,012	325	2.7	1.11 (0.99–1.24)		1.05 (0.92–1.20)
	Heavy	Non	5,428	166	7.1	1.40 (1.20–1.63)		1.34 (1.12–1.59)
		Mild–Moderate	35,336	434	2.8	1.11 (1.00–1.22)		1.05 (0.93–1.19)
		Heavy	64,024	698	2.5	1.06 (0.98–1.14)		1 (Ref.)
**Alzheimer's disease**								
Non	Non	Non	1,308,226	28,812	5.0	1 (Ref.)		1 (Ref.)
		Mild–Moderate	86,706	1,026	2.7	0.99 (0.94–1.07)		0.99 (0.93–1.06)
		Heavy	2,649	61	5.3	1.70 (1.32–2.19)		1.70 (1.32–2.19)
	Mild–Moderate	Non	87,657	1,185	3.1	0.99 (0.93–1.05)		0.99 (0.93–1.05)
		Mild–Moderate	114,312	809	1.6	0.83 (0.77–0.89)		0.82 (0.77–0.89)

© 2023 Jeon KH et al. *JAMA Network Open.*

Alcohol consumption status			Subjects (N)	Events (N)	IR (per 1000 PYs)	Non-drinkers as a reference		Sustained drinking at the same level as a reference
2009	2011	2013				HR (95% CI)		HR (95% CI)
		Heavy	6,416	70	2.5	1.06 (0.84–1.35)		1.06 (0.84–1.35)
	Heavy	Non	2,187	70	7.4	1.64 (1.29–2.09)		1.65 (1.29–2.10)
		Mild–Moderate	6,723	91	3.1	1.31 (1.06–1.61)		1.31 (1.06–1.61)
		Heavy	5,974	75	2.9	1.14 (0.90–1.44)		1.14 (0.90–1.44)
Mild–Moderate	Non	Non	121,702	2,097	3.9	1.09 (1.04–1.14)		1.47 (1.39–1.57)
		Mild–Moderate	89,422	836	2.1	0.91 (0.84–0.97)		1.24 (1.14–1.34)
		Heavy	5,032	87	4.0	1.36 (1.09–1.69)		1.82 (1.46–2.27)
	Mild–Moderate	Non	116,783	1,433	2.8	1.031(0.98–1.09)		1.39 (1.31–1.49)
		Mild–Moderate	667,076	3,147	1.1	0.72 (0.70–0.76)		1 (Ref.)
		Heavy	49,112	332	1.5	0.99 (0.89–1.11)		1.36 (1.21–1.53)
	Heavy	Non	5,831	118	4.7	1.31 (1.09–1.58)		1.75 (1.45–2.11)
		Mild–Moderate	49,487	351	1.6	0.93 (0.84–1.04)		1.27 (1.13–1.42)
		Heavy	30,559	233	1.7	0.96 (0.84–1.10)		1.30 (1.14–1.50)
Heavy	Non	Non	4,789	155	7.5	1.51 (1.29–1.77)		1.52 (1.26–1.83)
		Mild–Moderate	6,908	136	4.5	1.35 (1.13–1.61)		1.36 (1.11–1.65)
		Heavy	4,437	77	4.0	1.30 (1.04–1.63)		1.30 (1.02–1.65)
	Mild–Moderate	Non	7,506	191	5.9	1.62 (1.40–1.88)		1.64 (1.39–1.95)
		Mild–Moderate	64,843	474	1.7	0.94 (0.85–1.03)		0.94 (0.83–1.07)
		Heavy	28,012	252	2.1	1.10 (0.97–1.25)		1.11 (0.95–1.29)
	Heavy	Non	5,428	136	5.8	1.43 (1.21–1.70)		1.45 (1.20–1.76)
		Mild–Moderate	35,336	337	2.2	1.11 (0.99–1.24)		1.12 (0.97–1.29)
		Heavy	64,024	507	1.8	0.99 (0.91–1.09)		1 (Ref.)
**Vascular dementia**								
Non	Non	Non	1,308,226	3,309	0.6	1 (Ref.)		1 (Ref.)
		Mild–Moderate	86,706	134	0.4	0.98 (0.82–1.17)		0.99 (0.83–1.18)
		Heavy	2,649	11	1.0	1.85 (0.99–3.45)		1.90 (1.02–3.54)
	Mild–Moderate	Non	87,657	154	0.4	0.99 (0.84–1.17)		0.99 (0.85–1.18)
		Mild–Moderate	114,312	128	0.3	0.83 (0.69–1.00)		0.85 (0.71–1.03)
		Heavy	6,416	18	0.6	1.71 (1.07–2.73)		1.76 (1.10–2.81)
	Heavy	Non	2,187	6	0.6	1.09 (0.49–2.42)		1.10 (0.49–2.46)
		Mild–Moderate	6,723	9	0.3	0.70 (0.35–1.40)		0.72 (0.36–1.44)
		Heavy	5,974	14	0.5	1.31 (0.78–2.23)		1.35 (0.80–2.30)
Mild–Moderate	Non	Non	121,702	288	0.5	1.17 (1.03–1.32)		1.44 (1.24–1.68)
		Mild–Moderate	89,422	136	0.3	1.02 (0.85–1.21)		1.21 (1.01–1.47)
		Heavy	5,032	15	0.7	1.54 (0.93–2.57)		1.80 (1.08–3.01)
	Mild–Moderate	Non	116,783	180	0.4	0.91 (0.78–1.06)		1.10 (0.92–1.30)

© 2023 Jeon KH et al. *JAMA Network Open.*

Alcohol consumption status						Non-drinkers as a reference		Sustained drinking at the same level as a reference
2009	2011	2013	Subjects (N)	Events (N)	IR (per 1000 PYs)	HR (95% CI)		HR (95% CI)
		Mild–Moderate	667,076	658	0.2	0.87 (0.79–0.96)		1 (Ref.)
		Heavy	49,112	70	0.3	1.11 (0.87–1.43)		1.27 (0.99–1.63)
	Heavy	Non	5,831	16	0.6	1.09 (0.65–1.85)		1.32 (0.78–2.25)
		Mild–Moderate	49,487	71	0.3	1.01 (0.79–1.29)		1.16 (0.90–1.49)
		Heavy	30,559	40	0.3	0.92 (0.67–1.27)		1.06 (0.76–1.46)
Heavy	Non	Non	4,789	19	0.9	1.33 (0.84–2.12)		1.04 (0.63–1.73)
		Mild–Moderate	6,908	16	0.5	1.02 (0.61–1.69)		0.79 (0.46–1.35)
		Heavy	4,437	18	0.9	1.67 (1.02–2.73)		1.27 (0.75–2.13)
	Mild–Moderate	Non	7,506	29	0.9	1.59 (1.08–2.33)		1.22 (0.80–1.86)
		Mild–Moderate	64,843	100	0.4	1.13 (0.92–1.38)		0.88 (0.67–1.15)
		Heavy	28,012	52	0.4	1.28 (0.97–1.69)		0.99 (0.71–1.37)
	Heavy	Non	5,428	20	0.9	1.40 (0.89–2.20)		1.05 (0.64–1.72)
		Mild–Moderate	35,336	61	0.4	1.11 (0.86–1.45)		0.86 (0.63–1.18)
		Heavy	64,024	124	0.4	1.30 (1.07–1.57)		1 (Ref.)

Abbreviation: IR, Incidence rate; HR, hazard ratio; CI, confidence interval.

Adjusted for age, sex, smoking status, physical activity, area of residence, income, BMI, hypertension, diabetes mellitus, dyslipidemia, systolic blood pressure, fasting glucose level, total cholesterol level, and serum creatinine.

© 2023 Jeon KH et al. *JAMA Network Open.*

eFigure 1. Flow Chart of Study Population

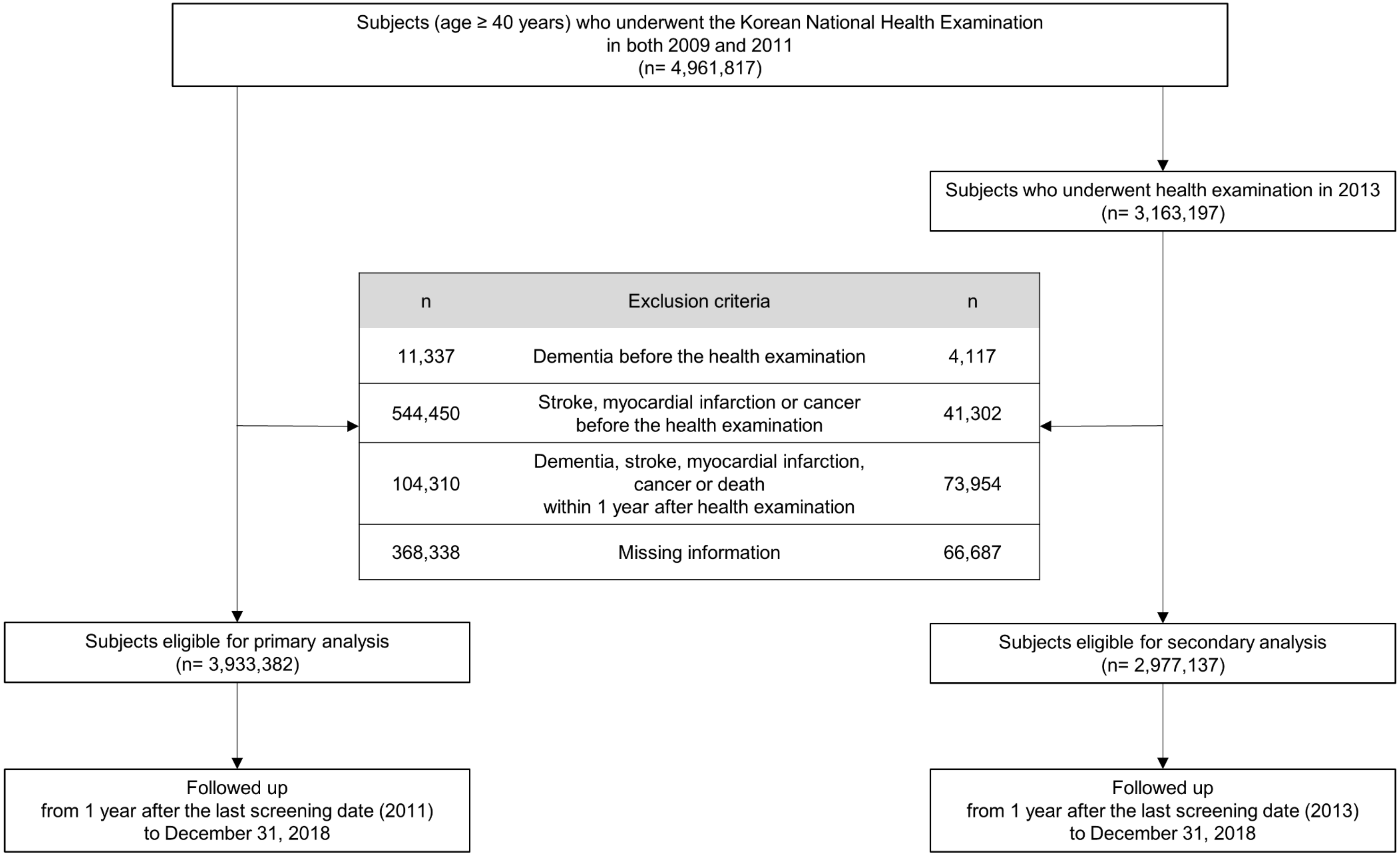

© 2023 Jeon KH et al. *JAMA Network Open.*

eFigure 2. Directed Acyclic Graph (DAG) Illustrating the Assumptions About the Causal Relationship Between Changes in Alcohol Consumption and Dementia

(A)

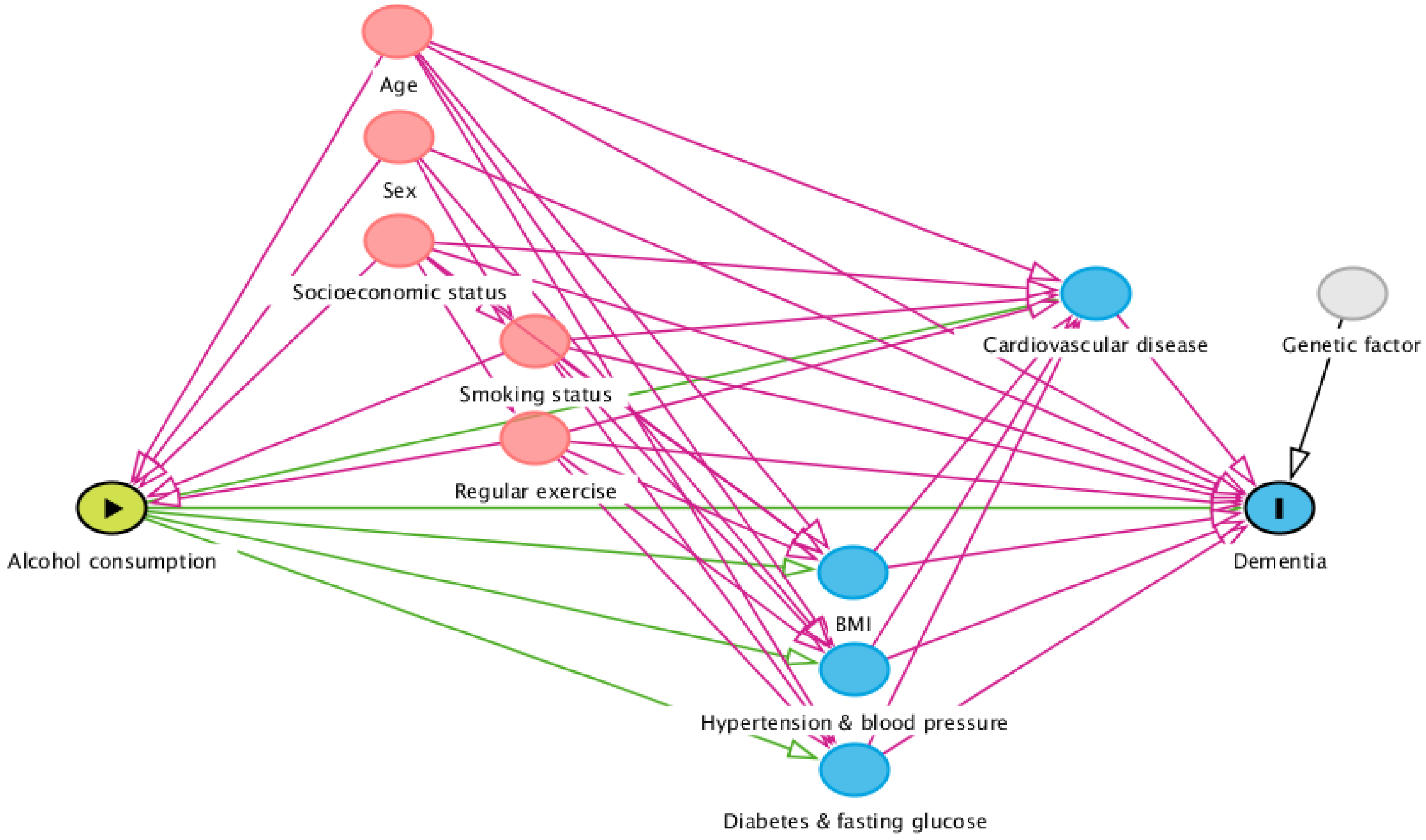

© 2023 Jeon KH et al. *JAMA Network Open.*

(B)

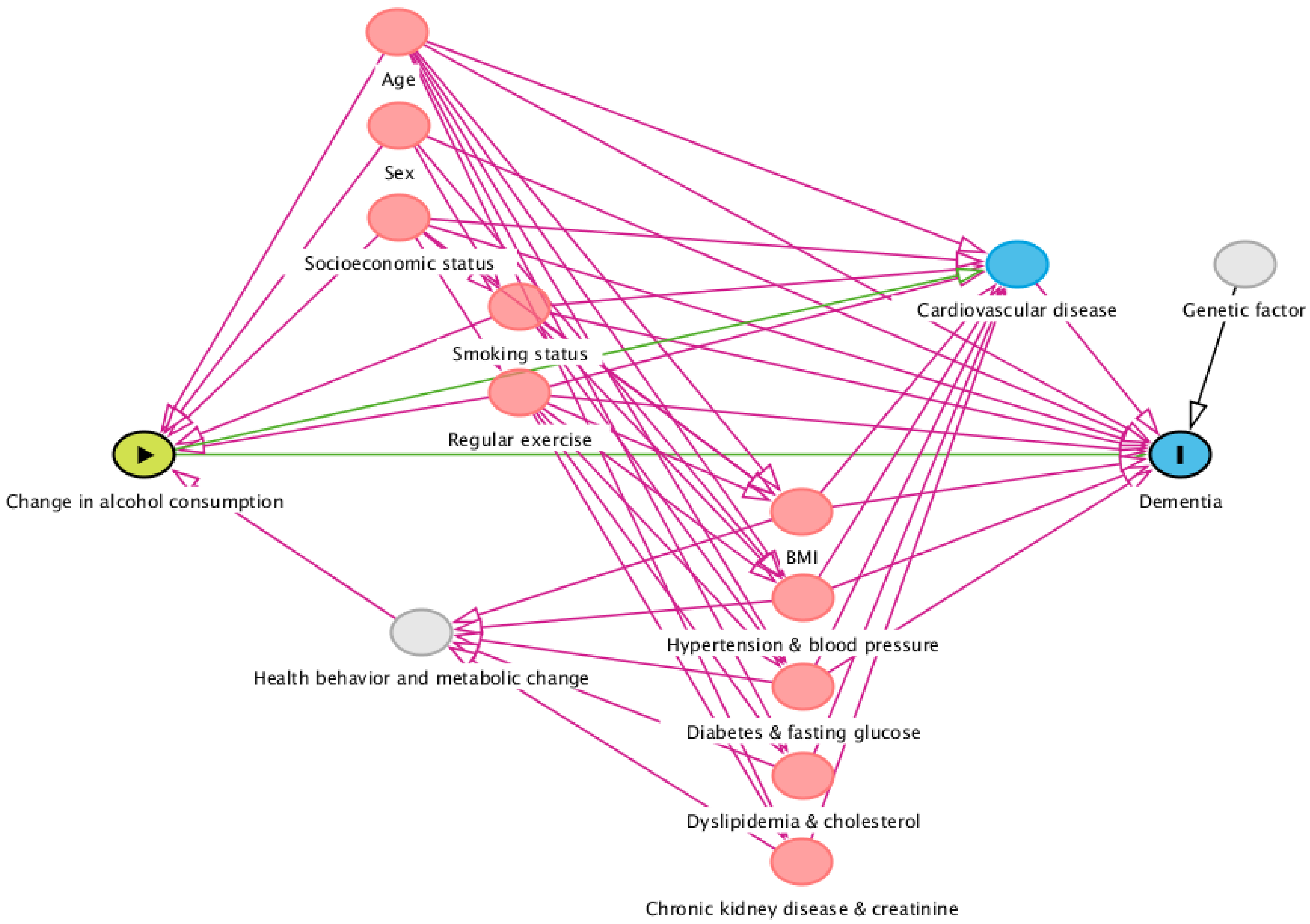

© 2023 Jeon KH et al. *JAMA Network Open.*

(A) Assumptions about the causal relationship between alcohol consumption and dementia; (B) Assumptions about the causal relationship between changes in alcohol consumption and dementia. The green circle with an arrow represents exposure; the blue circle with the I represents outcome; the other blue circle represents the ancestors of outcome (i.e., mediators); the red circles represent ancestors of exposure and outcome (i.e., confounders); the pale grey circles represent unobserved variables. The DAGs were constructed using the online software DAGitty, available at https://www.dagitty.net (accessed on 19 November 2022).

© 2023 Jeon KH et al. *JAMA Network Open.*
