# Supplementary material for: Changes in Alcohol Consumption and Risk of Dementia in a Nationwide Cohort in South Korea
Source: JAMA Netw Open. 2023 Feb 6;6(2):e2254771. doi: 10.1001/jamanetworkopen.2022.54771 (PMC12549098; doi:10.1001/jamanetworkopen.2022.54771)
Supplement: Supplement 2. — Data Sharing Statement [file jamanetwopen-e2254771-s002.pdf]

Data Sharing Statement

Jeon. Changes in Alcohol Consumption and Risk of Dementia in A Nationwide Cohort in South Korea. *JAMA Netw Open*. Published February 06, 2023.
doi:10.1001/jamanetworkopen.2022.54771

Data
Data available: No
